# Genome wide screening and comparative genome analysis for Meta-QTLs, ortho-MQTLs and candidate genes controlling yield and yield-related traits in rice

**DOI:** 10.1186/s12864-020-6702-1

**Published:** 2020-04-10

**Authors:** Bahman Khahani, Elahe Tavakol, Vahid Shariati, Fabio Fornara

**Affiliations:** 1 Department of Plant Genetics and Production, College of Agriculture, Shiraz University, Shiraz, Iran; 2 NIGEB Genome Center, National Institute of genetic Engineering and Biotechnology, Tehran, Iran; 3 Department of Biosciences, University of Milan, Via Celoria 26, 20133 Milan, Italy

**Keywords:** Breeding, MQTLs, Synteny analysis, yield-components

## Abstract

**Background:**

Improving yield and yield-related traits is the crucial goal in breeding programmes of cereals. Meta-QTL (MQTL) analysis discovers the most stable QTLs regardless of populations genetic background and field trial conditions and effectively narrows down the confidence interval (CI) for identification of candidate genes (CG) and markers development.

**Results:**

A comprehensive MQTL analysis was implemented on 1052 QTLs reported for yield (YLD), grain weight (GW), heading date (HD), plant height (PH) and tiller number (TN) in 122 rice populations evaluated under normal condition from 1996 to 2019. Consequently, these QTLs were confined into 114 MQTLs and the average CI was reduced up to 3.5 folds in compare to the mean CI of the original QTLs with an average of 4.85 cM CI in the resulted MQTLs. Among them, 27 MQTLs with at least five initial QTLs from independent studies were considered as the most stable QTLs over different field trials and genetic backgrounds. Furthermore, several known and novel CGs were detected in the high confident MQTLs intervals. The genomic distribution of MQTLs indicated the highest density at subtelomeric chromosomal regions. Using the advantage of synteny and comparative genomics analysis, 11 and 15 ortho-MQTLs were identified at co-linear regions between rice with barley and maize, respectively. In addition, comparing resulted MQTLs with GWAS studies led to identification of eighteen common significant chromosomal regions controlling the evaluated traits.

**Conclusion:**

This comprehensive analysis defines a genome wide landscape on the most stable loci associated with reliable genetic markers and CGs for yield and yield-related traits in rice. Our findings showed that some of these information are transferable to other cereals that lead to improvement of their breeding programs.

## Background

Rice (*Oryza sativa* L.) is the first global staple food and a genetically well-studied model crop for cereals [1, 2]. Grain weight (GW), tiller number (TN) and plant height (PH) are the major contributors to yield (YLD) in rice [1, 3, 4]. Heading date (HD) is also tightly associated with YLD and adaptation to different environments [3, 5–7]. Therefore, these traits are continuously targeted in breeding programs for producing new high-yielding varieties [8]. Since these traits are governed by several genes named as quantitative trait loci (QTLs) [2, 9], dealing with them is a challenge. QTL mapping provides accurate deciphering of genomic regions regulating these complex traits [10] and it has accelerated the success of breeders for improving quantitative traits by marker-assisted selection (MAS) [11]. However, the main problem faced by researchers in using QTL results are their dependency upon the population genetic backgrounds and the phenotyping environment that limit their applications in a wider range of populations or environments [10, 12].

Meta-analysis of QTLs unravels consensus and stable QTLs by merging different QTLs from independent experiments regardless of their genetic backgrounds, population types, evaluated locations and years [12–14]. Therefore, the Meta-QTL, with the abbreviation of “MQTL” in the rest of the manuscript, results are highly reliable and they can be widely used in breeding programs. Moreover, MQTL analysis consistently refines the position of QTLs and narrows down the confidence intervals (CI) that leads to accuracy of MAS [15, 16]. This conceptual approach has been used to detect MQTLs for various traits in barley [16, 17], wheat [11, 18–20], soybean [21, 22] and maize [15, 23–27]. In rice, there are two MQTL studies on YLD, PH and TN traits. Of these, one was conducted on 11 QTL studies published from 1998 to 2008 [28], whereas another was performed on 35 QTL studies covering the period of 1995 to 2006 [29]. Moreover, Daware et al. (2017) reported seven MQTLs related to GW from 7 QTL studies published only since 2008 to 2015 on *indica* and aromatic rice accessions [10].

We conducted a large and comprehensive meta-analysis on QTLs of YLD, TN, GW, PH and HD traits that are reported from 101 studies published from 1996 to 2018 in 122 bi-parental populations evaluated under unstressed conditions. It is the most comprehensive MQTL study for aforementioned traits in cereals and the first MQTL study on HD in rice. Beside MQTL study, each of the detected MQTLs was investigated to identify candidate genes (CGs) related to the evaluated traits. In addition, due to high synteny among rice, barley and maize [30, 31], we expanded our analysis to detect ortho-MQTLs in among these cereals. The uncovered novel MQTLs, ortho-MQTLs and candidate genes will aid genetic dissection of yield-related traits to improve yield in cereals.

## Results

### Main features of yield-related QTL studies in rice

**Fig. 1.**
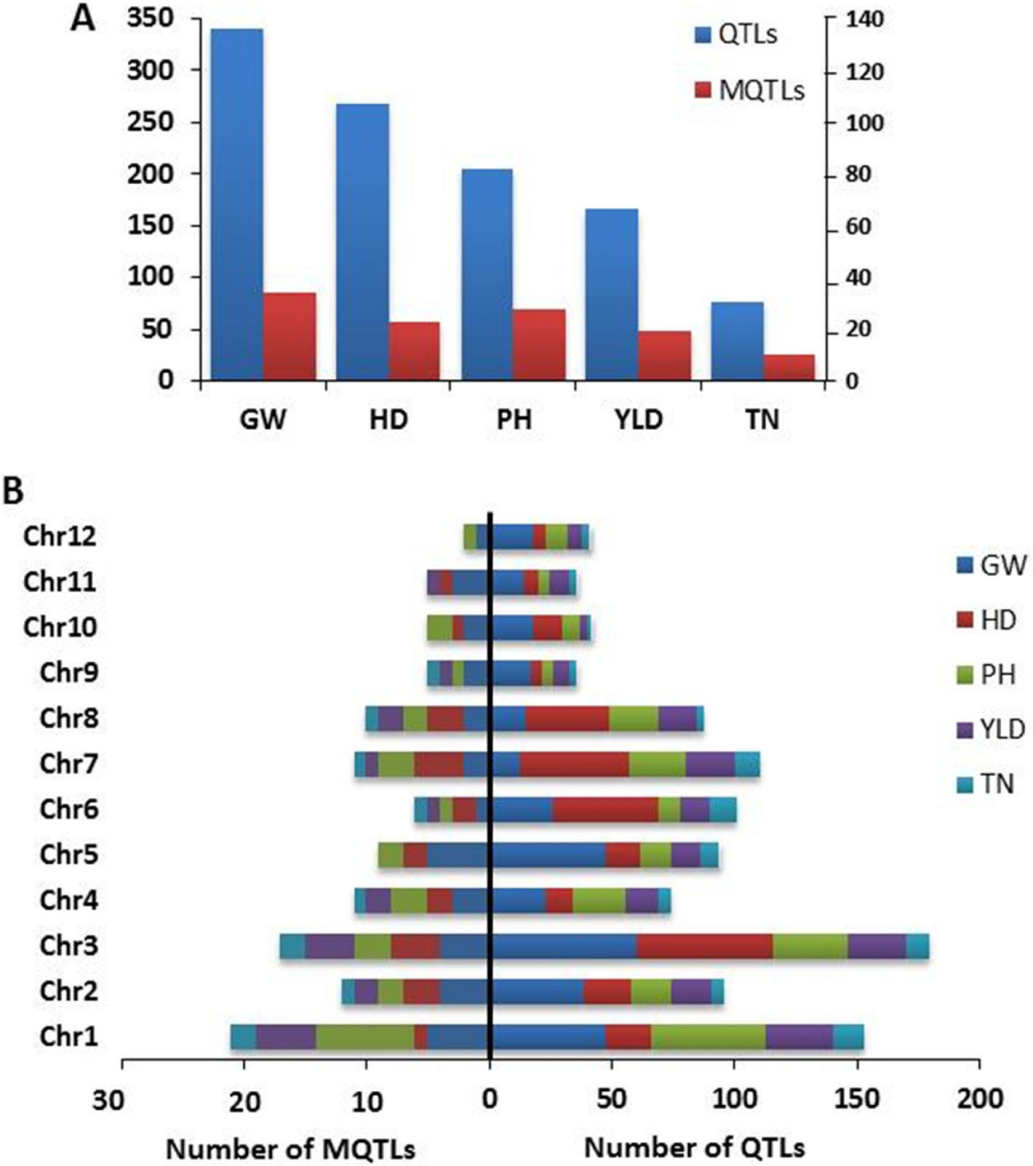
**a** Number of initial QTLs and MQTLs for YLD, HD, PH, GW and TN traits under normal condition (**b**) the distribution of QTLs and MQTLs on the twelve chromosomes in rice.

A total of 1052 QTLs controlling YLD, GW, HD, PH and TN in rice under unstressed conditions were retrieved from 122 populations reported in 101 studies since 1996 (Table 1). The number of QTLs for each trait and their distribution on 12 chromosomes of rice are presented in Fig. 1a and b. The QTLs scattered unevenly on different chromosomes; while chromosome 3 harbored the largest number of QTLs with 180 QTLs, followed by chromosome 1 (153 QTLs) and 7 (111 QTLs), chromosome 9 had the lowest number of QTLs with 36 QTLs.

**Table 1:** Summary of QTL studies used in the QTL meta-analysis for YLD, GW, HD, PH, and TN traits in rice under unstressed condition.

Ref No.	Number of QTL Population(s)	Parents of Population	Population Type	Population Size	No. of markers	Map density (cM)	Trait(s)	Reference
1	2	Tesanai 2 × CB	F2	171	44	14.12	GW	[32]
		Waiyin 2 × CB	F2	171	50	13.48	GW	
2	1	Zhai-Ye-Qing 8 × Jing-Xi 17	DH	132	106	13.37	HD, PH, GW	[33]
3	1	Palawan × IR42	F2	231	39	20.28	PH, GW, TN	[34]
4	1	Nipponbare × Kasalath	F2	186	343	1.11	HD	[35]
5	1	Zhenshan 97 × Minghui 63	F2	250	1167	1.11	YLD, GW, TN	[36]
6	1	Tesanai 2 × CB	F2	171	62	15.40	PH, GW	[37]
7	1	Nipponbare × Kasalath	BC	98	676	1.04	HD	[38]
8	1	IRGC 105491 × V20A	BC	300	101	10.93	YLD, GW, PH, HD	[39]
9	1	Nipponbare × Kasalath	BC	100	504	0.63	HD	[40]
10	1	Nipponbare × Kasalath	F2	296	373	0.64	HD	[41]
11	1	Zhenshan 97 × Minghui 63	F2	250	97	12.68	YLD, GW, TN	[42]
12	1	Miara × C6	DH	151	34	16.47	PH, HD, TN	[43]
13	1	ZYQ8 × JX17	DH	127	151	8.33	GW, HD, PH	[44]
14	1	ZYQ8 × JX17	RIL	107	48	9.91	HD, PH	[45]
15	1	Akihikari × Koshihikari	DH	212	495	0.58	HD	[46]
16	1	Nipponbare × Kasalath	BC	98	3266	0.46	YLD, PH, HD	[47]
17	1	Koshihikari × Kasalath	BC	187	39	11.85	HD	[48]
18	1	Nipponbare × Kasalath	BC	96	278	0.59	HD	[49]
19	1	RS-16 × BG90-2	BC	96	122	9.70	YLD, HD, PH, GW, TN	[50]
20	1	Reiho × Yamada-nishiki	DH	91	39	20.29	GW	[51]
21	1	Zhenshan 97 × Minghui 63	RIL	240	146	9.82	YLD, GW, TN	[52]
22	1	Zhenshan 97 × Minghui 63	RIL	240	166	10.98	YLD, GW, TN	[53]
23	1	Zenshan 97B × Milyang 46	RIL	209	124	7.72	YLD, GW	[54]
24	1	IR64 × Azuenca	DH	125	421	2.86	PH, GW	[55]
25	1	Johnson × Dora Lake Cross	F2	172	286	3.63	PH, HD, TN	[56]
26	1	IR64 × IRGC 105491	BC	400	123	12.78	YLD, GW, PH, HD	[57]
27	2	Jefferson × IRGC 105491	BC	258	153	10.13	YLD, GW, HD	[58]
		Jefferson × IRGC 105491	BC	353	153	10.13	GW, HD	
28	1	IAC165 × Co39	RIL	125	87	10.56	PH, TN	[59]
29	1	Lemont × Teqing	RIL	254	73	10.87	HD, PH	[60]
30	1	IR64 × Azuenca	DH	125	421	2.86	YLD, GW, HD	[61]
31	1	CT9993-5-10-1-M × IR62266-42-6-2	DH	220	399	5.49	YLD, HD, PH	[62]
32	1	Zhenshan 97 × Minghui 63	RIL	240	204	9.10	YLD, GW, TN	[63]
33	1	Milyang23 × Akihikari	RIL	191	182	6.56	TN	[64]
34	1	Zhenshan 97 × Minghui 63	RIL	240	214	7.82	PH	[65]
35	1	CT9993-5-10-1-M × IR62266-42-6-2	DH	220	182	4.19	YLD, HD, PH	[66]
36	1	IR36 × Nekken 2	BC	143	128	2.21	GW	[67]
37	1	Zhenshan 97 × Minghui 63	RIL	241	101	9.13	YLD, GW, TN	[68]
38	1	ZenShan 97B × IRAT109	RIL	187	339	2.99	YLD, GW	[69]
39	3	Lemont × Teqing	RIL	254	156	10.70	HD, PH	[70]
		Lemont × Teqing	BC	172	156	10.70	HD, PH	
		Lemont × Teqing	BC	177	156	10.70	HD, PH	
40	1	IR58025A × IC22015	BC	251	54	16.44	YLD, PH, GW, TN	[71]
41	1	Nipponbare × Kasalath	BC	98	3215	0.26	HD	[72]
42	1	B5 × Minghui 63	RIL	187	5441	0.29	YLD, GW, HD, PH	[73]
43	1	Moritawase × Koshihikari	RIL	92	22	11.47	HD	[74]
44	1	IR58821 × IR 52561	RIL	148	231	5.43	YLD, GW, PH, HD	[75]
45	1	Zenshan 97 × HR5	RIL	190	54	0.44	PH, HD	[76]
46	1	Guichao 2 × DXCWR	BC	159	52	11.57	YLD, GW	[77]
47	1	CL16 × IRGC 80470	F2	304	34	1.72	PH, TN	[78]
48	1	Lemont × Teqing	RIL	258	148	9.43	YLD, GW, PH, HD	[79]
49	1	H143 × Dongjinbyeo	F2	1009	10	11.16	HD	[80]
50	6	Nona Bokra × Koshihikari	F2	147	651	0.62	HD	[81]
		Nona Bokra × Koshihikari	BC	90	1216	0.72	HD	
		Nona Bokra × Koshihikari	BC	100	1216	0.72	HD	
		Nona Bokra × Koshihikari	BC	91	1216	0.72	HD	
		Nona Bokra × Koshihikari	BC	100	1216	0.72	HD	
		Nona Bokra × Koshihikari	BC	83	1216	0.72	HD	
51	1	Wuyunjing 8 × Nongken 57	DH	128	20	4.42	PH	[82]
52	1	Vandana × Way Rarem	F2	436	112	12.37	YLD, PH, HD	[83]
53	1	Milyang23 × Gihobyeo	RIL	164	505	1.58	YLD, GW, HD	[84]
54	1	IR71033-121-15 × Junambyeo	F2	146	73	12.37	GW, HD, TN	[85]
55	2	Hayamasari × Kasalath	F2	198	343	1.11	HD	[86]
		Hoshinoyume × Kasalath	F2	197	264	0.98	HD	
56	1	CT9993-5-10-1-M × IR62266-42-6-2	DH	220	207	4.96	YLD, HD, PH	[87]
57	2	Nipponbare × Koshihikari	BC	79	21	8.50	HD	[88]
		Nipponbare × Koshihikari	BC	127	21	10.09	HD	
58	1	Suweon365 × Chucheongbyeo	RIL	231	347	2.50	YLD, HD	[89]
59	1	Chunjiang × TN1	DH	120	99	9.75	HD	[90]
60	1	Norungan × IR64	RIL	93	126	7.61	YLD, GW, PH, TN	[91]
61	1	IR20 × Nootripathu	RIL	250	24	14.90	PH, TN	[92]
62	1	Nipponbare × W630	F2	141	721	0.72	HD	[93]
63	2	Nipponbare × IR1545-339	F2	301	1937	0.72	HD	[94]
		TK8 × IR1545-339	F2	304	1937	0.72	HD	
64	2	Minghui 63 × Teqing	RIL	190	185	0.63	HD	[95]
		Zenshan 97 × Teqing	RIL	190	185	0.63	HD	
65	1	CT9993-5-10-1-M × IR62266-42-6-2	DH	135	399	5.49	YLD, HD, GW, PH, TN	[5]
66	1	Nanyangzhan × Chuan 7	RIL	185	141	9.92	PH, HD, GW	[96]
67	1	9311 × Nipponbare	RIL	150	SNP	SNP	GW, HD, PH, TN	[97]
68	1	Minghui 63 × Zenshan 97	RIL	241	SNP	SNP	GW	[98]
69	1	Zenshan 97 × 9311	BC	244	2030	0.74	GW, PH	[99]
70	3	XieqingzaoB × Zhonghui9308	BC	176	2030	0.74	YLD, PH, GW	[100]
		XieqingzaoB × Zhonghui9308	RIL	226	2030	0.74	GW, HD, TN	
		XieqingzaoB × Zhonghui9308	BC	185	2030	0.74	YLD, HD, GW	
71	1	Pusa1266 × Jaya	RIL	310	121	21.95	YLD, GW, PH, HD	[3]
72	1	Teqing × Binam	BC	77	718	2.49	YLD, GW, PH	[101]
73	2	SLG × Zenshan 97	RIL	102	83	2.45	GW	[102]
		M53 × SLG	F2	957	83	2.45	GW	
74	2	Tarom Molaei × Teqing	BC	85	718	2.49	YLD, GW	[103]
		Tarom Molaei × IR64	BC	72	718	2.49	YLD, GW	
75	1	Guanghui 116 × LaGrue	RIL	307	58	18.36	YLD, GW, TN	[104]
76	1	Xieqingzao B × R9308	RIL	215	45	8.72	PH	[105]
77	1	R1128 × Nipponbare	F2	781	SNP	SNP	PH	[106]
78	1	Xiaobaijingzi × Kongyu 131	RIL	220	73	12.89	YLD, PH	[107]
79	1	Kaybonnetlpa1-1 × Zhe733	RIL	255	52	13.27	PH, HD	[108]
80	1	IR55419-04/2 × TDK1	BC	365	418	0.68	YLD, HD, PH	[109]
81	1	Big Grain1 × Xiaolijing	RIL	269	95	9.76	HD, GW	[110]
82	2	Bengal × PSR-1	RIL	198	2030	0.74	PH, GW	[111]
		Cypress × PSR-1	RIL	174	2030	0.74	PH	
83	1	M201 × JY293	RIL	234	32	8.73	GW	[112]
84	1	Xian80 × Suyunuo	F2	175	2030	0.74	PH, HD	[113]
85	1	9311 × Peiai 64	RIL	132	SNP	SNP	YLD	[114]
86	1	Gang46B × K1075	RIL	182	11	5.71	GW	[115]
87	1	YTH288 × IR66215-44-2-3	F2	167	235	0.67	HD	[116]
88	1	IR36 × Pokkali	F2	113	6	7.5	GW	[117]
89	1	9311 × W2014	RIL	131	SNP	SNP	PH, GW, YLD	[118]
90	1	TS × H193	RIL	191	SNP	SNP	GW, HD	[119]
91	1	Swarna × IRGC81848	BC	94	62	18.19	YLD, PH, HD, TN	[4]
92	1	Nanyangzhan × Zenshan 97B	RIL	190	443	2.42	GW	[120]
93	1	Yuexiangzhan × Shengbasimiao	RIL	186	394	0.72	YLD	[121]
94	1	Nipponbare × Kasalath	F2	139	343	0.73	HD	[122]
95	1	Francis × R998	RIL	213	SNP	SNP	GW, YLD	[123]
96	1	Cocodrie × Vandana	F2	187	136	7.75	YLD	[124]
97	1	Cocodrie × N-22	RIL	181	SNP	SNP	TN	[125]
98	1	PR114 × IRGC104433	BC	185	SNP	SNP	GW	[126]
99	2	CSSL39 × 9311	F2	1024	185	0.63	HD	[127]
		CSSL39 × 9311	F2	846	185	0.63	HD	
100	2	Bengal × PSR-1	RIL	198	2030	0.74	HD	[128]
		Cypress × PSR-1	RIL	174	2030	0.74	HD	
101	2	D123 × Shennong265	BC	178	40	12.24	GW, PH, HD	[129]
		D123 × Shennong265	BC	314	29	19.04	YLD, GW, PH, TN	

*BC* Backcross, *DH* Double Haploids, *RIL* Recombinant Inbred Lines, *YLD* Yield, *GW* Grain Weight, *PH* Plant Height, *HD* Heading Date, *TN* Tiller Number

The number of QTLs was varied in different evaluated quantitative traits. Among the studied traits, GW and HD had the highest number of QTLs with 339 and 267 QTLs, respectively, followed by PH, YLD and TN with 204, 165 and 77 QTLs, respectively (Fig. 1b). The QTLs for GW were mainly located on chromosome 3, 5 and 1 with 60, 48 and 48 QTLs, respectively, and the majority of QTLs for HD were placed on chromosomes 3 (56), 7 (44) and 6 (43). Consistently with previous reports [28, 130], chromosome 1 had the highest number of QTLs for YLD. Chromosome 1 also harbored the highest number of QTLs for PH and TN traits (Fig. 1b).

### Detected MQTLs for yield-related traits

**Fig. 2:**
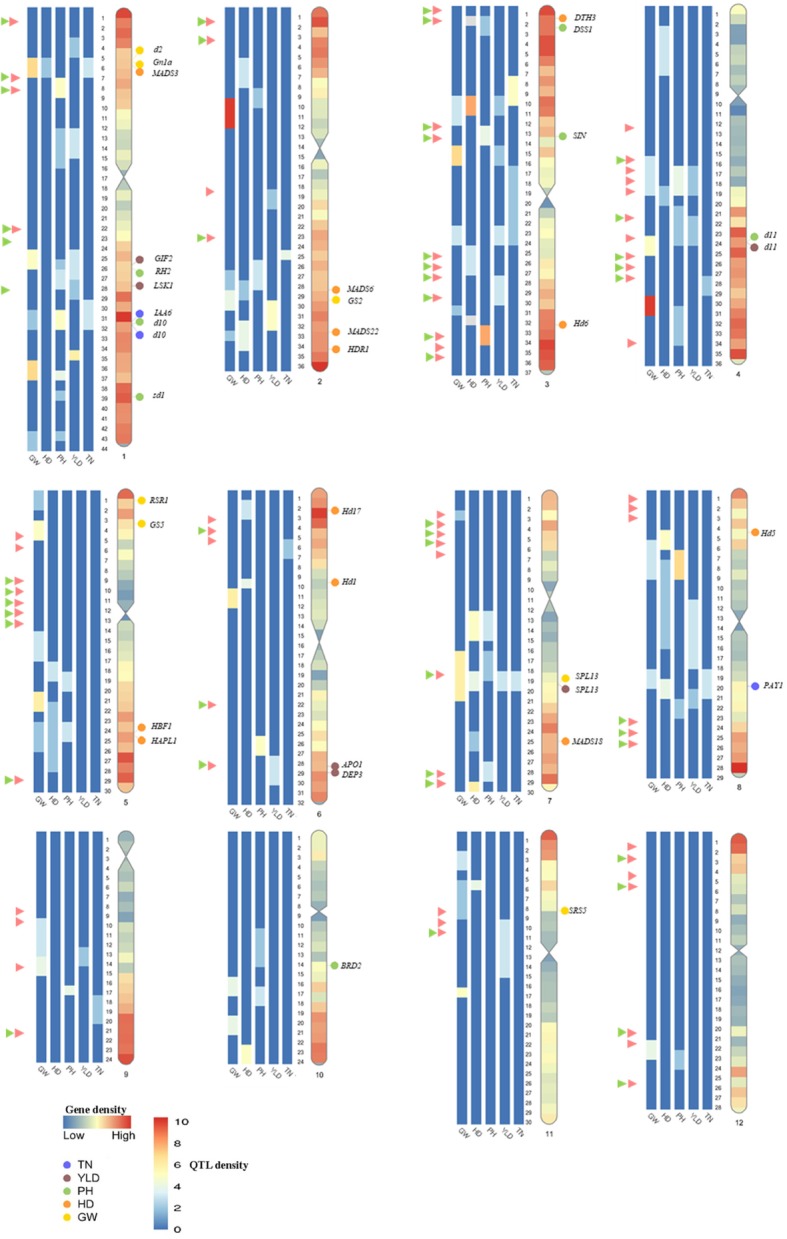
Heatmap of MQTLs for YLD, GW, PH, HD, and TN. The position of MQTLs on the rice genome are presented in Mb. The gene density is indicated on the right chromosome. The proved genes located at each MQTL interval are presented on the chromosome. The red and green head arrows indicate the selective sweep regions and the functional variants on coding regions, respectively. For additional information on the position of initial QTLs present in a MQTL see additional file 1.

A total of 960 QTLs out of the 1052 QTLs (91%) from 122 populations were successfully projected on the reference map (Table 2). The MQTL analysis confined these QTLs into 114 MQTLs (11.87 %) with QTLs originated from at least two studies for all the aforementioned traits (Table 3; Fig. 1, 2 and S1). Of these MQTLs, 58 MQTLs (50.8 %) were obtained from at least three independent studies (Table 3; Additional file 1).

**Table 2:** The number of initial QTLs on the 12 chromosomes of rice for YLD, GW, HD, PH, and TN traits under unstressed condition used for MQTL analysis after integrating into the reference map.

Chromosome	YLD	GW	HD	PH	TN	Total
1	26	48	18	44	13	149
2	17	38	18	15	5	93
3	20	59	54	28	8	169
4	12	22	11	20	3	68
5	9	42	12	12	8	83
6	12	26	40	9	10	97
7	16	12	43	20	10	101
8	15	15	24	17	3	74
9	7	16	5	4	2	34
10	3	13	10	6	2	34
11	8	12	6	5	3	34
12	3	10	2	7	2	24
Total	148	313	243	187	69	960

*YLD* Yield, *GW* Grain Weight, *PH* Plant Height, *HD* Heading Date, *TN* Tiller Number

**Table 3:** Summary of the detected MQTLs for YLD, GW, HD, PH, and TN traits in rice under unstressed condition

Trait	Chr.	MQTL	Flanking markers	Position on the consensus reference map (cM)	Confidence interval (cM)	Genomic position on the rice genome (Mb)	Number of initial QTLs	Number of studies	Number of Populations	Number of genes laying at the MQTL interval	References^a^
GW	1	MQTL-GW1	RM3233-C52458s	32.27	3.73	5.05-6.58	7	5	5	175	
	1	MQTL-GW2	RM3366-RM1349	103.77	2.67	24.26-25.07	5	3	4	108	
	1	MQTL-GW3	RM1095-RM5914	129.95	2.01	30.92-31.50	2	2	2	77	
	1	MQTL-GW4	RM3447-RM6618	144.29	3.07	35.25-37.01	7	4	4	221	
	1	MQTL-GW5	RM8049-RM6831	178.53	3.35	42.07-43.17	2	2	2	165	
	2	MQTL-GW6	RM452-G243A	49.85	5.97	9.56-11.75	10	6	7	165	
	2	MQTL-GW7	RM7245-RM221	110.97	6.42	26.44-27.60	2	2	2	147	
	2	MQTL-GW8	R2216-RM5993	124.58	2.79	28.41-29.70	4	3	3	171	
	2	MQTL-GW9	RM8030-RM5958	140.25	1.06	32.48-32.83	2	2	2	48	
	3	MQTL-GW10	R134-RM4512	46.9	5.21	9.49-11.30	3	3	3	271	
	3	MQTL-GW11	RM6931-C11260S	70.66	2.07	14.98-15.47	7	4	4	37	
	3	MQTL-GW12	S1466-RM6425	92.12	3.12	22.98-23.82	3	2	2	59	[10]
	3	MQTL-GW13	R2462-R63525	136.1	0.8	30.10-30.38	2	2	2	37	
	4	MQTL-GW14	RM5687-RM6314	34.77	15.5	15.74-18.44	3	2	2	136	
	4	MQTL-GW15	R278-RM2848	74.44	0.4	23.43-24.49	5	4	4	158	[10]
	4	MQTL-GW16	R2737-RM5503	97.98	4.41	29.15-30.17	10	4	4	139	
	5	MQTL-GW17	S2309-S2136	11.47	3.27	0.94-1.29	2	2	2	47	
	5	MQTL-GW18	RM7349-RM3322	30.98	2.01	3.24-4.26	5	3	3	106	
	5	MQTL-GW19	S21985S-E2801S	60.92	6.32	14.54-16.95	3	2	2	181	
	5	MQTL-GW20	RM6282-E10316S	80.4	5.48	20.24-21.13	6	3	3	103	
	5	MQTL-GW21	RG470-RM3620	102.11	3.59	23.48-25.20	2	2	2	204	
	6	MQTL-GW22	R10069S-RM3330	59.06	2.81	10.46-11.06	6	5	5	47	
	7	MQTL-GW23	RM5100+RM5752	10.75	2.19	2.21-2.56	2	2	2	23	
	7	MQTL-GW24	R646-RM1048	64.86	11.35	16.96-20.16	6	5	5	261	
	8	MQTL-GW25	S12665S-C1251S	58.89	5.8	5.80-8.15	3	2	2	139	
	8	MQTL-GW26	S3680-RM8264	80.09	6.78	18.25-19.83	3	3	3	128	
	9	MQTL-GW27	C1454-C397	78.8	7.33	9.63-12.28	3	3	3	169	
	9	MQTL-GW28	S4677S-RM7039	92.53	1.96	13.62-14.68	4	3	3	107	
	10	MQTL-GW29	RM6144-RM3229	40.14	6.1	15.60-16.69	4	3	3	101	
	10	MQTL-GW30	RM7300-RM147	61.67	2.23	19.93-20.94	4	2	2	140	
	11	MQTL-GW31	RM1812-RM1124	22.71	7.29	2.40-3.85	3	2	2	155	
	11	MQTL-GW32	S20163S-RM3701	38.66	11.35	5.37-8.10	2	2	2	244	
	11	MQTL-GW33	R10329S-RM4746	69.14	0.86	16.04-16.57	5	2	2	29	
	12	MQTL-GW34	RM3326-C11001SA	77.56	6.92	21.74-22.45	4	3	3	35	
HD	1	MQTL-HD1	C12072S-C52458	31.9	4.1	5.51-6.58	2	2	2	128	
	2	MQTL-HD2	E50474S-RM3505	32.82	6.81	5.64-7.54	3	2	2	212	
	2	MQTL-HD3	C1236-R418	117.41	8.35	27.36-28.94	2	2	2	180	
	2	MQTL-HD4	R685-RG256	134.61	1.2	31.26-33.93	4	4	4	363	
	3	MQTL-HD5	C51477S-RM6013	5.78	1.85	1.03-1.66	11	8	11	79	
	3	MQTL-HD6	C68-RM6496	44.5	2.18	9.31-10.14	8	5	5	130	
	3	MQTL-HD7	RM5626-RM7097	104.68	10.87	24.86-26.87	3	2	2	196	
	3	MQTL-HD8	R2404-RM3867	142.69	0.59	31.38-31.74	13	8	11	61	
	4	MQTL-HD9	R2811-RM4835	12.69	8.62	2.08-6.98	3	3	3	225	
	4	MQTL-HD10	RM6314-S10644	42.81	10.76	18.44-19.04	2	2	2	52	
	5	MQTL-HD11	S2467-RM3969	69.48	7.81	17.14-18.93	3	2	2	169	
	5	MQTL-HD12	E60663S-R1714	99.43	26.84	21.14-27.80	2	2	2	874	
	6	MQTL-HD13	C425A-RM5218	8.38	2.6	1.64-2.36	3	3	3	112	
	6	MQTL-HD14	RM6836-RM8238	54.49	0.14	9.30-9.45	4	3	3	13	
	7	MQTL-HD15	RM214-RM7183	50.66	0.3	12.78-14.95	5	5	5	97	
	7	MQTL-HD16	RM432-RM7087	65.58	0.3	18.95-19.35	4	4	4	29	
	7	MQTL-HD17	C50171S-RM478	88.85	4.48	24.62-25.94	2	2	2	158	
	7	MQTL-HD18	S11279-C924	116.89	0.05	29.01-29.21	6	4	5	31	
	8	MQTL-HD19	E60560S-RZ562	51.31	1.95	4.17-5.42	5	4	4	112	
	8	MQTL-HD20	RM3181-RM7027	65.87	9.82	7.55-15.84	2	2	2	439	
	8	MQTL-HD21	RM8264-RM4668	84.77	1.18	19.83-20.53	4	4	4	59	
	10	MQTL-HD22	RM496-RM590	68.87	2	22.43-23.04	5	4	4	82	
	11	MQTL-HD23	S20162S-RM6894	36.24	3.59	5.37-5.91	4	4	4	60	
PH	1	MQTL-PH1	RM5359-RM6630	41.15	6.65	7.17-8.36	5	3	3	152	
	1	MQTL-PH2	C1905-E3004S	72.04	5.86	12.64-15.16	2	2	2	184	
	1	MQTL-PH3	R2374-RM3475	107.61	2.84	25.06-26.04	2	2	2	99	
	1	MQTL-PH4	RM5461-V176	115.3	1.4	26.90-27.11	3	2	2	25	
	1	MQTL-PH5	C1459-RM3411	129.19	2.12	30.53-31.31	5	5	5	117	
	1	MQTL-PH6	RM8278-RM6618	146.15	0.07	36.62-37.01	4	3	3	36	
	1	MQTL-PH7	RM3442-RM8235	150.9	3.2	38.20-38.43	2	2	2	40	
	1	MQTL-PH8	RM8049-E60152S	176.16	6.28	42.07-42.68	2	2	2	95	[29]
	2	MQTL-PH9	RM6853-RM452	44.24	5.59	8.95-9.56	2	2	2	39	
	2	MQTL-PH10	S13984-RM599	107.09	5.32	25.62-27.10	3	3	3	186	
	3	MQTL-PH11	RM6013-R2247	9.16	3.62	1.66-2.48	2	2	2	125	
	3	MQTL-PH12	RM7249-RM6080	61.25	2.77	12.90-13.93	4	4	4	82	
	3	MQTL-PH13	C831-S851	147.69	0.65	32.92-33.03	8	7	7	25	
	4	MQTL-PH14	S10983-RM6314	36.41	1.18	16.77-18.44	4	2	2	82	
	4	MQTL-PH15	C2043-RM3839	67.33	11.14	20.56-23.90	2	2	2	428	[29]
	4	MQTL-PH16	G379B-RZ879B	108.46	4.29	30.63-33.12	2	2	2	359	
	5	MQTL-PH17	R1436-RZ649	72.97	4.71	18.25-19.54	3	3	3	127	[29]
	5	MQTL-PH18	RM3476-R3802S	101.7	2.48	23.84-24.60	3	2	2	107	
	6	MQTL-PH19	RM5371-RM6782	98.23	0.64	25.82-26.04	5	4	4	26	
	7	MQTL-PH20	RM214-RM7183	50.65	0.3	12.78-14.95	3	2	2	97	
	7	MQTL-PH21	RM1135-RM5405	60.21	4.05	16.93-18.58	2	2	2	120	
	7	MQTL-PH22	RM3555-RM5720	107.11	1.89	27.89-28.66	3	3	3	123	
	8	MQTL-PH23	E20920S-C1107	60.6	5.56	6.03-8.68	7	5	6	164	
	8	MQTL-PH24	RM7356-RM210	92.21	1.7	21.28-22.47	2	2	2	101	[29]
	9	MQTL-PH25	RM1189-RM7048	103.29	3.16	16.27-16.93	4	3	3	80	
	10	MQTL-PH26	RM3311-RM8201	22.39	6.64	10.62-13.76	2	2	2	204	[29]
	10	MQTL-PH27	RM5304-S11014	45.45	8.06	16.34-17.98	3	3	3	164	
	12	MQTL-PH28	C11001SA-R10289S	82.7	7.6	22.45-23.06	2	2	2	60	
YLD	1	MQTL-YLD1	RG246-T96	21.31	8.24	3.50-4.44	2	2	2	122	[28]
	1	MQTL-YLD2	C1905-C45	71.67	5.43	12.64-14.79	3	3	3	154	[28]
	1	MQTL-YLD3	RM5919-RM3475	106.72	6.72	24.73-26.04	3	3	3	146	[28]
	1	MQTL-YLD4	RM7414-RM3336	120.29	5.52	27.17-28.61	2	2	2	192	
	1	MQTL-YLD5	RM8061-RM6950	139.01	0.03	34.12-34.50	6	5	5	44	
	2	MQTL-YLD6	RM7413-RM8254	69.29	11.41	18.45-19.74	2	2	2	132	[28, 29]
	2	MQTL-YLD7	RM6933-RM3857	128.89	8.94	29.30-31.84	5	3	3	264	[29]
	3	MQTL-YLD8	S13802-C2184A	44.79	3.92	9.24-10.39	2	2	2	183	
	3	MQTL-YLD9	C1186-G144	68.71	2.3	14.55-15.33	2	2	2	71	[28]
	3	MQTL-YLD10	RM5864-RZ403	90.64	3	22.39-23.08	3	3	3	49	[28, 29]
	3	MQTL-YLD11	S10209-S11669	127.52	3.48	27.82-29.55	3	3	3	205	
	4	MQTL-YLD12	E30341S-RM471	32.96	8.12	16.28-18.82	2	2	2	152	
	4	MQTL-YLD13	RM3337-RM3839	69.02	7.93	21.73-23.90	2	2	2	310	[29]
	6	MQTL-YLD14	E4392S-RM439	109.26	4.78	27.37-29.62	3	3	3	275	[29]
	7	MQTL-YLD15	RM432-RM2966	66.59	4.89	18.95-19.73	3	3	3	65	[28]
	8	MQTL-YLD16	E31128S-E2623S	70.14	5.57	11.58-17.51	3	3	3	258	
	8	MQTL-YLD17	G1073A-RG1	89.53	4.71	20.66-21.64	2	2	2	93	
	9	MQTL-YLD18	C397-S1824	85.91	4.14	12.28-13.63	2	2	2	98	[29]
	11	MQTL-YLD19	S10207-R120	59.93	8.03	9.06-14.95	3	3	3	247	[28, 29]
TN	1	MQTL-TN1	RM522-C52458	31.76	6.52	5.24-6.58	3	2	2	158	
	1	MQTL-TN2	RM3614-S10712	127.12	2.46	29.82-31.36	3	3	3	247	
	2	MQTL-TN3	C41-RM6617	98.74	1.17	24.52-24.76	4	4	4	28	
	3	MQTL-TN4	RM1022-C68	39.95	5.93	7.23-9.31	5	5	5	257	
	3	MQTL-TN5	C60318S- RM6425	87.26	12.69	16.78-23.82	2	2	2	475	[29]
	4	MQTL-TN6	S733-R738	90.92	5.95	27.86-28.90	2	2	2	119	
	6	MQTL-TN7	RM253-RG213	27.32	2.97	5.42-6.28	2	2	2	100	
	7	MQTL-TN8	RM432-RM2966	66.38	4.44	18.95-19.73	3	2	2	65	
	8	MQTL-TN9	RM5767-RM1578	84.18	10.44	18.81-20.97	3	3	3	182	
	9	MQTL-TN10	RM6543-C11503S	115.41	11.29	17.75-19.88	2	2	2	317	

*YLD* Yield, *GW* Grain Weight, *PH* Plant Height, *HD* Heading Date, *TN* Tiller Number, *Chr* chromosome

^a^The rice MQTLs with reference indicates that this MQTL was also reported in the previous studies

The number of MQTLs for each trait was distributed unevenly among rice chromosomes. In this analysis 34, 23, 28, 19 and 10 MQTLs were detected for GW, HD, PH, YLD and TN traits, respectively. The distribution of MQTLs for each trait on each chromosome is presented in table 3 and Additional file 1. The most of the MQTLs associated with GW were located on chromosomes 1 and 5, whereas MQTLs of HD were mainly located on chromosomes 3 and 7 (Table 3). Overall, we could detect at least one MQTL for GW on all of the chromosomes (Table 3). Apparently, chromosome 1 was predominantly involved in controlling PH, YLD and TN traits. The lowest MQTLs for GW, HD, PH, YLD and TN were mainly located on chromosomes 5, 9, 10, 11 and 12. In general, there was a positive correlation between QTLs density and the number of MQTLs on chromosomes for all studied traits (r=0.90, Table 2 and 3, Fig. 1b). Moreover, the traits with the higher number of QTLs had the higher number of MQTLs (Fig. 1a).

A MQTL with the higher number of initial QTLs is a more stable MQTL independent from genetic background and environment. MQTL-HD8 with 13 initial QTLs had the highest number of QTLs derived from 11 different populations followed by MQTL-HD5, MQTL-GW6 and MQTL-GW16 with 11, 10 and 10 initial QTLs derived from 11, 7 and 4 different populations, respectively (Table 3). These MQTLs appeared as the most robust, viable and stable QTLs in different locations and years. Furthermore, we identified 22 overlapping MQTLs or clusters of MQTLs which controlled at least two traits (Additional file 1). Interestingly, two clusters of MQTLs located on chromosomes 7 and 8 includes all studied traits (Additional file 1). The overlapping MQTLs are likely to contain CGs with broad pleiotropic effects.

**Fig. 3:**
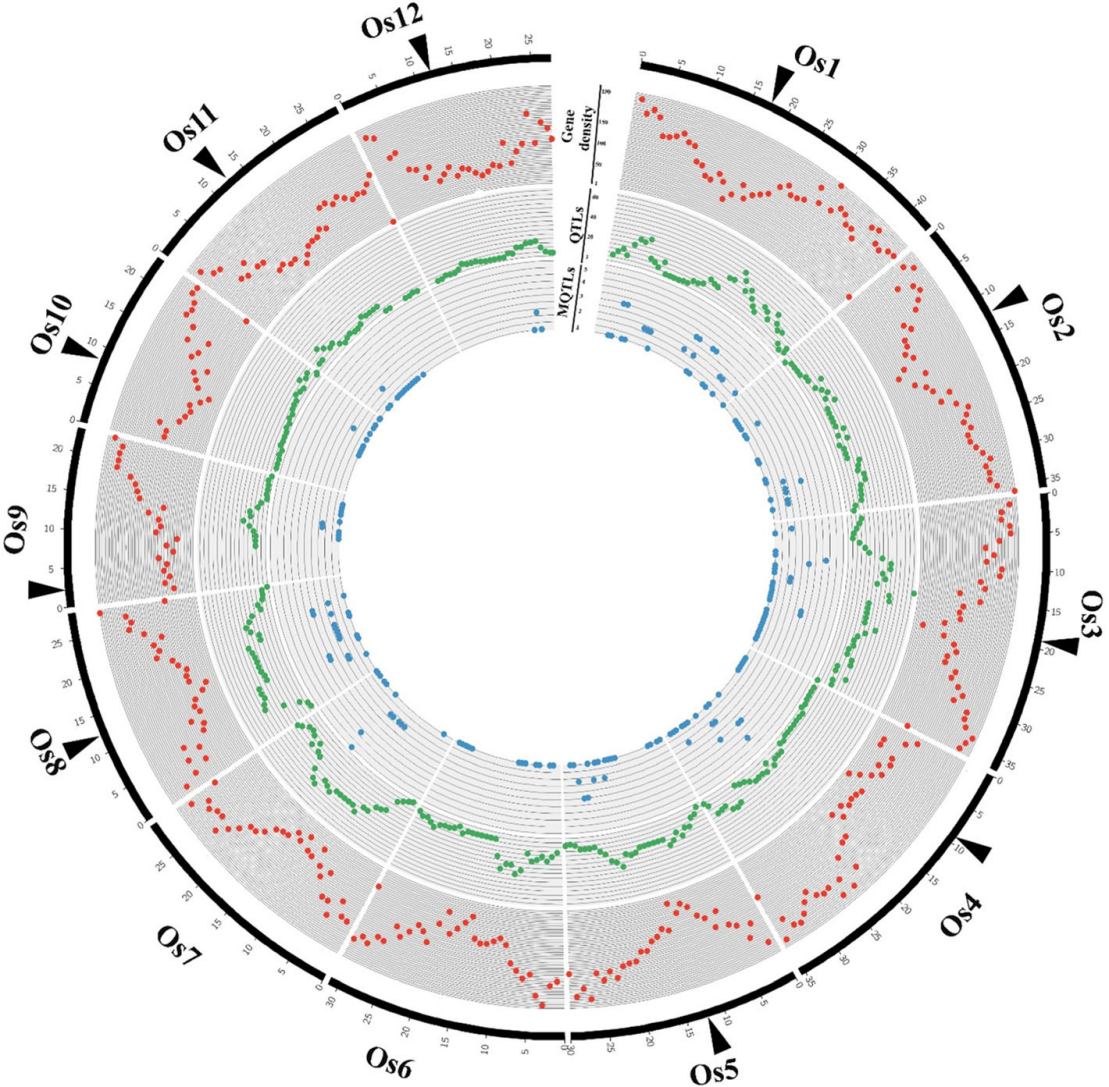
Distribution pattern of the gene density and the number of QTLs and MQTLs on the rice genome. The outermost circle represents the chromosomes position on the rice genome in Mb. The black head arrow indicates the centromeric position of the chromosome. The second circle with red color outlines the gene density on the rice genome. The third and fourth inner circles with green and blue colors display the number of QTLs and MQTLs, respectively.

The distribution pattern of MQTLs on the rice genome was investigated and compared with genomic events including selective sweep regions and gene density. The number of MQTLs per chromosome varied from 2 (chromosome 12) to 21 (chromosome 1) with an average of 9.5 MQTLs per chromosome (Table 3; Fig. 2 and additional file 1). The overview on the distribution of gene density on the rice genome revealed that sub-telomeric regions harbor most of the genes (Fig. 2 and 3). Similarly, the distribution of QTLs and MQTLs displayed comparable pattern to the gene density over the rice genome (Fig. 2 and 3). We detected the lowest number QTLs at the centromeric intervals for all studied traits (Fig. 2 and 3).

A total of 23 and 12 MQTLs were co-located on the selective sweep regions and the regions containing known functional variants on the rice genome, respectively [131]. These regions can be further investigated among the rice genetic resources for improving yield in breeding programs (Fig. 2, Additional file 3).

### Detected candidate genes for yield-related traits

An advantage of MQTL analysis is to confine the CI that it consequently results in increasing the precision of CGs prediction. The MQTL analysis reduced the average CI up to 3.5 folds with an average of 4.85 cM in MQTLs in compared to the mean CI of the original QTLs. Among the detected MQTLs, the CI in 13 MQTLs (MQTL-GW13, GW15, GW33, HD8, HD14, HD15, HD16, HD18, PH6, PH13, PH19, PH20 and YLD5) was reduced to < 1 cM (Table 3). For instance the CI was reduced to 0.63, 0.35, 0.15 and 0.71 Mb in compare to their initial QTLs interval of 4.77, 3.03, 2.31 and 3.96 Mb in MQTL-HD5, HD8, HD14 and YLD15, respectively. Consequently, the number of genes in their interval was limited to 79, 61, 13 and 65 genes, in compare to initial 737, 456, 156 and 309 genes in the original QTLs interval, respectively. The confined interval in MQTL-HD5, HD8, HD14 and YLD15 contain *DTH3*, *Hd6*, *Hd1* and *OsSPL13* well-known genes, respectively, controlling aforementioned traits (Additional file 2). All the annotated genes located at each MQTL interval and the potential candidate genes based on their function are reported in additional file 2. Among the annotated genes in each MQTL interval, the following well-known proved genes controlling HD (*Hd1*, *Hd5*, *Hd6*, *Hd17*, *HBF1¸ HAPL1, DTH3, HDR1, OsMADS3, OsMDAS6, OsMADS18* and *OsMADS22*), GW (*d2*, *Gn1a*, *d11*, *GS2*, *RSR1*, *GS5*, *OsSPL13* and *SRS5*), PH (*d10*, *sd1*, *d11, OsRH2, OsDSS1, OsSIN* and *BRD2*), YLD (*GIF2*, *OsLSK1*, *APO1*, *d11* and *DEP3*) and TN (*OsIAA6, d10* and *PAY1*) were identified. The putative novel CGs for each trait were reported in Additional file 2 and discussed in more details here.

### MQTLs and CGs for Grain Weight

GW is one of the fundamental yield components with a notable capability for boosting YLD in rice. GW QTLs are consistently introduced as a highly substantial objective for breeding programs [132]. In our study, a high number of GW QTLs (339) were analyzed (Fig. 1); that resulted in detection of 34 MQTLs. The identified MQTLs were distributed on all the rice chromosomes including five MQTLs on chromosomes 1 and 5, four MQTLs on chromosomes 2 and 3, three MQTLs on chromosomes 4 and 11, two MQTLs on chromosomes 7, 8, 9 and 10 and one MQTLs on chromosomes 6 and 12 (Table 3). The MQTL-GW16 and MQTL-GW6 are considered as the most stable QTLs with 10 QTLs (Table 3). The following remarkable cloned genes that effectively control GW such as *d2*, *Gn1a, GS2, d11, RSR1, GS5, OsSPL13* and *SRS5* [1, 132–135] were located at MQTL-GW1, GW8, GW15, GW17, GW18, GW24 and GW32 intervals, respectively in which MQTL-GW5, GW18 and GW24 were co-located with selective sweep regions (Additional file 2 and additional file 3).

Beside known genes, we identified novel CGs based on their annotated function that are presented in Additional file 2 and potentially can be a regulator of GW. In MQTL-GW6 on chromosome 2, the *Os02g0283800* annotated as *OsBAK1-5* or *OsSERK2* regulates grain size and number [136]. The *OsALMT7* gene located at MQTL-GW7 interval was shown to affect grain size [137]. In MQTL-GW10 interval on chromosome 3, *OsEZ1* and *NRL2* genes contribute to the grain size and YLD in rice [138, 139]. Furthermore, MQTL-GW20 with six GW QTLs (Table 3) from three populations, contains the *Os05g0414700* gene encoding a BRASSINOSTEROID INSENSITIVE 1-associated receptor kinase 1 that might be a new CG for regulating GW. We also detected new gene which encodes brassinosteroid receptor kinase gene at MQTL-GW28 intervals that was recently shown to collocated at selective sweep regions and have high impact on development and therefore could be good candidates for further functional investigation.

### MQTLs and CGs for Heading Date

HD controlled by polygenes that substantially encompasses the seed production and YLD [7]. In this analysis, we could detect 23 MQTLs related to HD including four MQTLs on chromosomes 3 and 7, three MQTLs on chromosomes 2 and 8, two MQTLs on chromosomes 4, 5 and 6 and one MQTL on chromosomes 1, 10 and 11. Among them, MQTL-HD8 had the largest number of initial QTLs with 13 QTLs (Table 3). The cloned genes such as *OsMADS3*, *OsMADS6, HDR1*/*OsMADS22, DTH3*, *Hd6*, *HAPL1/HBF1*, *Hd17, Hd1*, *OsMADS18* and *Hd5* [6, 7, 140–144] controlling this trait were located on MQTL-HD1, HD3, HD4, HD5, HD8, HD12, HD13, HD14, HD17 and HD19 on chromosomes 1, 2, 3, 6, 7 and 8 (Additional file 2).

In addition to these genes, there were other promising CGs based on their confirmed function related to HD or floral formation that are highlighted in Additional file 2. We detected potential HD CGs at MQTL-HD12 interval including circadian clock genes. Among the genes in MQTLHD-9 region, the *ETR2* gene is reported to affect flowering time [145]. In MQTL-HD18, the *OsbZIP62* is shown to interact with Hd3a protein and affect flower in rice [143]. The *SDG701* gene at the same MQTL is also related to circadian elements [146]. The B-box (BBX) proteins in three MQTLs are key factors in photoperiodic mechanism [147]. Moreover, basic region/leucine zipper motif (bZIP), GF14 proteins, MADS-box, *FT-like* genes and F-box proteins are considered as a decisive and pervasive regulator in flowering pathways in rice that are present in some of detected MQTLs [6, 143, 148, 149].

### MQTLs and CGs for Plant height

PH is one of the leading attributes to Green Revolution introduced by semi-dwarf phenotype and using large amount of nitrogen fertilizer [1]. For PH trait, 28 MQTLs were identified on the all rice chromosomes except chromosome 11 comprising the highest number of MQTLs on chromosome 1 (8), three MQTLs on chromosomes 3, 4 and 7, two MQTLs on chromosomes 2, 5, 8 and 10 and one MQTL on chromosomes 6, 9 and 12 (Table 3). The MQTLPH-13 and MQTL-PH23 were the most stable ones that had the largest number of initial QTLs with 8 and 7 QTLs for PH, respectively. The proved cloned genes controlling PH were identified on the following MQTLs: *OsRH2* on MQTL-PH3 [150], *d10* on MQTL-PH5 [151], *sd1* on MQTL-PH7 [1], *OsDSS1* on MQTL-PH11 [152], *OsSIN* on MQTL-PH12 [153], *d11* on MQTL-PH15 [154] and *BRD2* on MQTL-PH26 [155].

Moreover, there were CGs related to PH which were located in these MQTLs intervals that were highlighted in Additional file 2. In MQTL-PH6, *WLP2* [156] and in MQTL-PH9, *OsYABBY4* gene [157] regulate PH through GA pathway. There is an encouraging finding that *Os03g0350100* and *Os03g0350300* located at MQTL-PH12 are similar to brassinosteroid receptor kinase (Additional file 2) which causes dwarf-phenotype in rice [1]. Additionally, the mutation in Trichome Birefringence-like proteins (TBL) located at MQTL-PH7, PH15 and PH28 reduces PH in rice [158].

### MQTLs and CGs for Yield

YLD is the most prominent criteria in the rice breeding programs [96]. We identified 19 MQTLs for YLD comprising five MQTLs on chromosome 1, four MQTLs on chromosome 3, two MQTLs on chromosomes 2, 4 and 8, one MQTL on chromosomes 6, 7, 9 and 11 (Table 3). The proved genes such as *GIF2* [159], *OsLSK1* [160], *d11* [154], *APO1* [161]/*DEP3* [9] and *OsSPL13* [135] controlling yield were located on MQTL-YLD3, YLD4, YLD13 YLD14 and YLD15 respectively, and most of them were co-located with selective sweep regions and functional variants on coding regions (Additional file 3) . The list of CGs is presented in Additional file 2. For instance, *Os01g0171000* gene at MQTL-YLD1 which encodes BRASSINOSTEROID INSENSITIVE 1-associated receptor kinase is a potential candidate for higher yield. In MQTL-YLD6, the *SWEET15* gene was reported to have substantially effects on YLD through regulating seed filling in rice [162].

### MQTLs and CGs for number of tillers

TN is a foremost feature in plant architecture and grain production in cereals. Despite its agronomic importance, only a few tiller controlling genes have been identified so far [163, 164]. A total of 10 MQTLs associated with TN are detected in our analysis including two MQTLs on chromosomes 1 and 3 and one MQTL on chromosomes 2, 4, 6, 7, 8 and 9 (Table 3). The cloned genes such as *OsIAA6* [165], *d10* [1] and *PAY1* [166] genes controlling TN trait were situated on MQTL-TN2, MQTL-TN2 and MQTL-TN9, respectively. The list of CGs is presented in Additional file 2. A homologous of *OsPIN5b* gene at MQTL-TN10 on chromosome 9 controls TN, PH, panicle size and other traits related to plant architecture [167]. Moreover, the *GSK3/SHAGGY-like kinase*, *GA2ox* genes on MQTL-TN1 and GRAS TFs on MQTL-TN4 and MQTL-TN7 are also reported to regulate TN [163].

### Ortho-MQTL mining in barley and maize

**Fig. 4:**
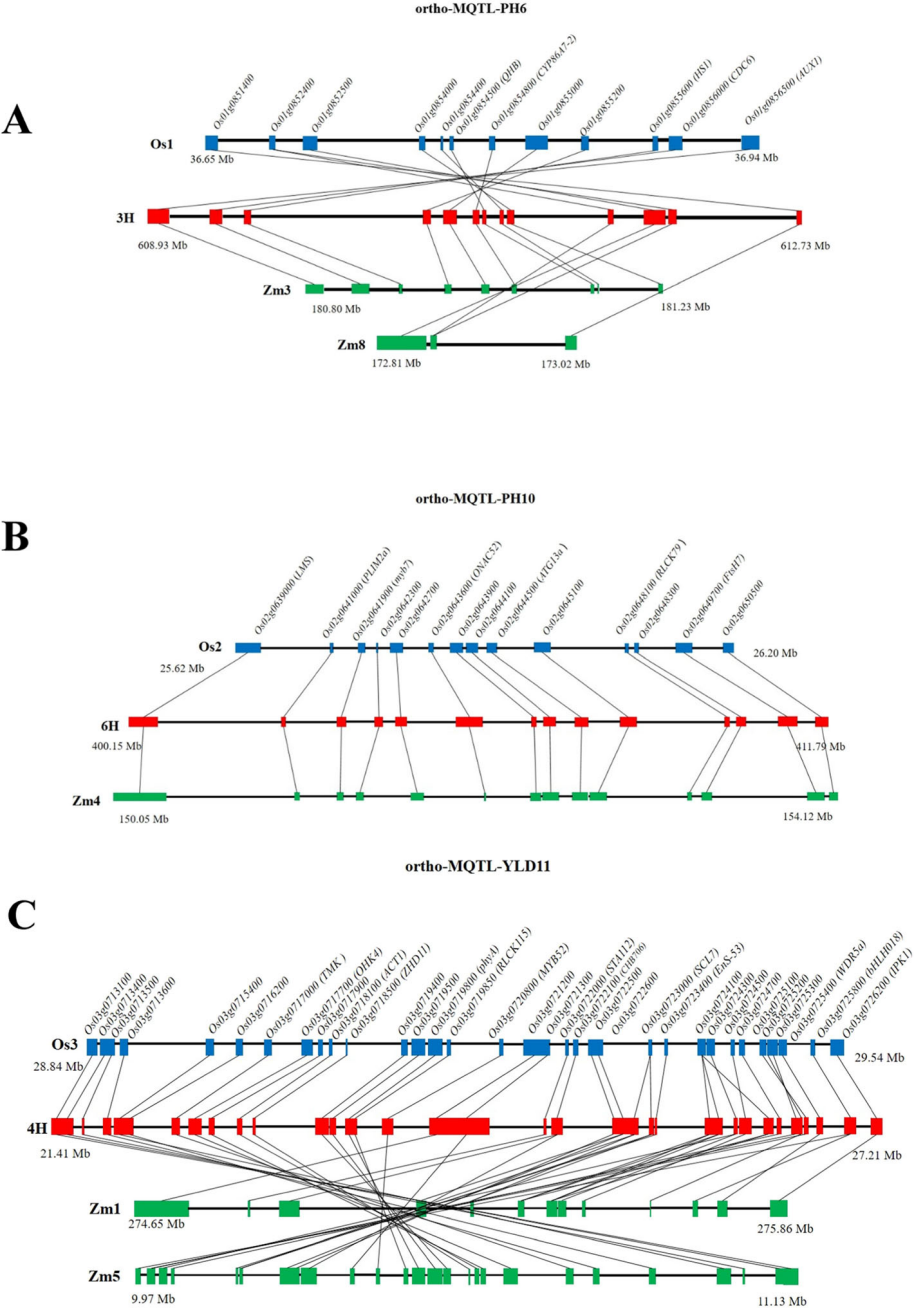
Syntenic region of MQTLs among rice, barley and maize. **a** Ortho-MQTL-PH6 indicates syntenic regions among identified PH MQTLs in rice (MQTL-PH6), barley (MQTL3H.1) and maize (MQTL40 and MQTL91), (**b**) Ortho-MQTL-PH10 indicates syntenic regions among identified PH MQTLs in rice (MQTL-PH10), barley (MQTL6H.4) and maize (MQTL47), (**c**) Ortho-MQTL-YLD11 indicates syntenic regions among identified YLD MQTLs in rice (MQTL-YLD11), barley (MQTL4H.2) and maize (MQTL10 and MQTL56). The chromosome number, genomic position and common genes among rice, barley and maize are indicated. More details are presented in Table 4 and Table S3.

Due to the high synteny among barley, maize and rice and also the economically importance of the studied traits in all cereals, 58 of the most reliable MQTLs derived from at least three independent studies in rice were selected for investigation of ortho-MQTLs in barley and maize. Consequently, a total of 11 ortho-MQTLs were detected for rice and barley including four ortho-MQTLs for HD, three for GW and PH and one for YLD. Moreover, a total of 15 ortho-MQTLs were identified in rice and maize consisting of nine and six ortho-MQTLs for YLD and PH, respectively. Among them, three ortho-MQTLs (ortho-MQTL-PH6, ortho-MQTL-PH10 and ortho-MQTL-YLD11) were cross-species in all the three crops (Table 4; Fig. 4).

**Table 4:** Ortho-MQTLs in barley and maize based on the syntenic region with MQTLs in rice

Ortho-MQTL	Rice MQTL	Rice chr. no. (genomic position in Mb)	Barley/Maize original MQTL name	Barley chr. no. (genomic position in Mb)	Maize chr. no. (genomic position in Mb)	Barley/Maize MQTL reference
ortho-MQTL-GW4	MQTL-GW4	1 (35.25-37.00)	MQTL3H.1	3 (608.93-624.91)	-	[17]
ortho-MQTL-GW10	MQTl-GW10	3 (9.49-10.37)	MQTL4H.3	4 (466.66-491.74)	-	[17]
ortho-MQTL-GW15	MQTL-GW15	4 (24.11-24.47)	MQTL2H.2	2 (605.54-612.53)	-	[17]
ortho-MQTL-HD6	MQTL-HD6	3 (9.31-10.12)	MQTL4H.3	4 (472.36-499.20)	-	[17]
ortho-MQTL-HD8	MQTL-HD8	3 (31.38-31.73)	MQTL5H.3	5 (602.26-606.03)	-	[17]
ortho-MQTL-HD13	MQTL-HD13	6 (1.88-2.03)	MQTL7H.2	7 (21.85-24.06)	-	[17]
ortho-MQTL-HD18	MQTL-HD18	7 (29.01-29.21)	MQTL2H.1	2 (38.13-41.95)	-	[17]
ortho-MQTL-PH17	MQTL-PH17	5 (18.31-18.87)	MQTL1H.2	1 (413.75-419.78)	-	[17]
ortho-MQTL-PH12	MQTL-PH12	3 (12.90-13.93)	MQTL8	-	1 (59.13-63.14)	[25]
ortho-MQTL-PH19	MQTL-PH19	6 (25.86-26.01)	MQTL96	-	9 (98.83-100.13)	[25]
ortho-MQTL-PH23	MQTL-PH23	8 (7.91-8.61)	MQTL46	-	4 (71.97-73.91)	[25]
		8 (6.03-8.64)	MQTL107		10 (62.30-69.48)	
ortho-MQTL-PH27	MQTL-PH27	10 (17.22-17.94)	MQTL57	-	5 (28.98-31.56)	[25]
ortho-MQTL-YLD2	MQTL-YLD2	1 (12.64-14.67)	MQTL44	-	8 (40.26-47.91)	[24]
			MQTL54			[26]
ortho-MQTL-YLD3	MQTL-YLD3	1 (25.79-26.03)	MQTL3.8	-	3 (218.26-218.71)	[23]
		1 (24.76-25.96)	MQTL88		8 (148.19-151.37)	[25]
			MQTL-58			[26]
ortho-MQTL-YLD7	MQTL-YLD7	2 (29.32-30.14)	MQTL22	-	4 (163.97-166.84)	[24]
			MQTL48			[25]
		2 (29.31-29.55)	MQTL5.8		5 (207.11-207.76)	[23]
			MQTL62			[25]
			MQTL-38			[26]
ortho-MQTL-YLD10	MQTL-YLD10	3 (22.53-23.09)	MQTL-6	-	1 (253.16-253.76)	[26]
			MQTL8		1 (259.49-260.63)	[24]
		3 (22.41-23.07)	MQTL29		5 (18.91-19.22)	[24]
			MQTL57			[25]
ortho-MQTL-YLD14	MQTL-YLD14	6 (28.96-29.53)	MQTL5.5	-	5 (54.42-58.71)	[23]
			MQTL-33			[26]
			MQTL58			[25]
		6 (28.96-29.57)	MQTL66		6 (89.31-91.27)	[25]
ortho-MQTL-YLD15	MQTL-YLD15	7 (19.04-19.68)	MQTL27	-	2 (208.53-209.56)	[25]
		7 (19.30-19.65)	MQTL41		7 (157.21-158.18)	[24]
ortho-MQTL-YLD16	MQTL-YLD16	8 (15.59-16.59)	MQTL7	-	1 (224.11-225.11)	[24]
ortho-MQTL-YLD19	MQTL-YLD19	11 (9.07-14.02)	MQTL23	-	2 (122.07-132.24)	[25]
ortho-MQTL-PH6	MQTL-PH6	1 (36.62-37.00)	MQTL3H.1	3 (608.93-612.89)	-	[17]
		1 (36.79-37.00)	MQTL40	-	3 (180.80-181.23)	[25]
		1 (36.63-36.72)	MQTL91		8 (172.81-173.23)	[25]
ortho-MQTL-PH10	MQTL-PH10	2 (25.62-26.46)	MQTL6H.4	6 (400.15-418.52)	-	[17]
		2 (25.62-26.23)	MQTL47	-	4 (150.05-154.32)	[25]
ortho-MQTL-YLD11	MQTL-YLD11	3 (28.81-29.55)	MQTL4H.2	4 (20.17-27.21)	-	[17]
		3 (29.26-29.54)	MQTL10	-	1 (274.65-275.86)	[24]
		3 (28.84-29.51)	MQTL56		5 (9.97-11.13)	[25]

*YLD* Yield, *GW* Grain Weight, *PH* Plant Height, *HD* Heading Date, *TN* Tiller Number, *Chr* chromosome

As a result, rice MQTL-HD8 and MQTL-HD18 on chromosomes 3 and 7 were in co-linear region with similar MQTLs on chromosome 5H (MQTL5H.3) and 2H (MQTL2H.1) in barley, respectively (Table 4). The rice MQTL-PH17 was in co-linear region with a MQTL for PH on chromosome 1H (MQTL1H.2) in barley (Table 4). Similarly, an ortho-MQTL for MQTL-YLD15 on rice chromosome 7 was detected on chromosome 2 and 7 in maize harboring MQTLs for yield (Table 4).

Moreover, three rice MQTLs (MQTL-PH6, MQTL-PH10 and MQTL-YLD11) on chromosomes 1, 2 and 3, respectively, were located in a syntenic region with detected MQTLs on barley chromosomes 3H, 6H and 4H, respectively, as well as maize MQTLs for YLD (Fig. 4a, b and c; Table 4). Ortho-MQTL mining could validate our analysis and it can facilitate detecting underlying regulatory genes with evolutionary history and conservative function. All the genes located at the ortho-MQTLs regions along with their annotations were reported in Additional file 4. Remarkably, the orthologous of well-known proved genes including *Hd6*, *OsbZIP62* and *OsSPL13* in rice were detected in barley and maize ortho-MQTLs.

## Discussion

### Distribution pattern of QTLs and MQTLs and identification of CGs

The investigated QTLs were not evenly distributed on all chromosomes of rice. Chromosomes 1, 3 and 7 harbor the largest number of QTLs that are in agreement with previous reports [2, 168]. Chromosomes 3, 1 and 5 harbored the largest number of QTLs associated with GW in parallel with preliminary results in rice [2]. Additionally, HD had the highest initial QTLs on chromosomes 3, 7 and 6 as shown by prior results [7].

The MQTL analysis detects the most stable QTLs regardless of the genetic background, the phenotyping variations in different places and years, and the markers density that are the main restrictions of QTL mapping [8, 10–12, 17]. Association mapping is another approach with higher accuracy in compare to QTL mapping for identification of genomic regions underlying quantitative traits [10]. But, this approach faces remarkable false-positive results due to the structure of population used in the analysis [10]. Therefore, MQTL analysis is considered as the most reliable approach to identify stable loci controlling quantitative traits. Here, MQTL analysis confined a total of 960 QTLs into 114 MQTLs on twelve chromosomes of rice for all studied traits. Chromosomes 1 and 12 with 21 and 2 MQTLs had the highest and the least number of MQTLs, respectively. Similarly, Swamy and Sarla (2011) reported the highest number of MQTLs for YLD on chromosomes 1, 2 and 3 [28]. Beside the common MQTLs reported in other studies [10, 28, 29] for YLD, PH, TN and GW (Table 3), we identified 31 new MQTLs for GW, 21 new MQTLs for PH, 9 new MQTLs for TN and 7 new MQTLs for YLD and this is the first MQTL study conducted on HD (Table 3).

It is hypothesized QTL density is chiefly related to gene density and polymorphism rate [15]. Our results demonstrated that the most of MQTLs and QTLs were located at the subtelomeric regions where gene density is relatively high. Previous investigations in barley and maize reported similar results in which QTLs and MQTLs were densely located at the subtelomeric regions [15, 17, 169].

The higher sequence polymorphism rate and functional variants at coding regions resulted in higher differentiation in allele frequencies in rice [131]. We detected 12 MQTLs which were precisely located at these regions. In addition, 23 MQTLs were cope with the selective sweep regions that occurred during the domestication processes and profoundly affected the selection and spread of critical traits [131]. These results provide beneficial information for breeders to proficiently apply diverse genetic resources for improving rice and other cereal crops. We detected fundamental genes for GW, HD PH, and YLD (Additional file 3) controlling aforementioned traits in these overlapping MQTLs.

Moreover, MQTL analysis considerably reduces the CI in compared to the initial QTLs. It consequently diminishes the number of genes anchoring at the QTL interval that are in agreement with previous reports [16, 23, 27, 130, 168]. Therefore, MQTL analysis enhances the precision of CGs prediction and the detection of markers for marker assistant selection in breeding [17]. In our analysis, the average CI was reduced up to 3.5 folds in compare to the mean CI of the original QTLs, therefore, the number of genes located at the QTLs interval was extensively reduced. Fundamental genes such as *Hd1*, *Hd5*, *Hd6*, *Hd17*, *DTH3, HDR1, OsMADS3, OsMDAS6, OsMADS18* and *OsMADS22* for HD, and *d2*, *Gn1a*, *d11*, *GS2*, *RSR1*, *GS5*, *OsSPL13* and *SRS5* for GW were still located at the narrowed MQTLs interval. Furthermore, among the limited number of genes annotated at each MQTL interval we detected potential CGs for PH, YLD and TN attributes that are listed in Additional file 2.

### Ortho-MQTL mining

Despite the high interest in identification of genes involved in YLD and yield-related traits in barley and maize as two economically important crops, the responsible genes have largely remained unknown due to their complex genomes. Given a close evolutionary relation among grass genomes [170], synteny analysis of barley and maize with rice as a model crop in grasses enabled us to broaden our genetic information among these species [30]. Identification of ortho-MQTLs among these close species expands their utility and it also validates their stability and the confidence of related CGs. Here we selected the most prospective rice MQTLs containing at least three QTLs from different studies to explore their conserved syntenic regions reported in similar MQTLs studies on the same traits in barley and maize to identify ortho-MQTLs (Table 4).

For rice MQTL-HD8 there is a MQTL in the syntenic region on barley [17] controlling ortho-MQTL-HD8 containing a rice *Hd6* orthologous gene (*HORVU5Hr1G097230*) known to have a high impact on HD [7]. *Moreover, in the syntenic region of rice MQTL-HD18 in barley there is a MQTL on chromosome 7 (ortho-MQTL-HD18) encompassing OsbZIP62* orthologous gene (*HORVU2Hr1G017020*) that regulates flowering in rice [143]. All other ortho-MQTLs identified between rice and barley are described in table 4 and the orthologous genes are presented in Additional file 4 among which of the most important CGs were explained during the corresponding rice MQTL discussion.

In maize, we detected 15 ortho-MQTLs for PH and YLD (Table 4; Additional file 4). Five ortho-MQTLs including ortho-MQTL-YLD2, ortho-MQTL-YLD3, ortho-MQTL-YLD7, ortho-MQTL-YLD10 and ortho-MQTL-YLD14 contained at least two MQTLs in maize reported in different studies (Table 4). In the syntenic region of rice MQTL-PH112 on chromosome 1 of maize (ortho-MQTL-PH12) there was an orthologous of rice *VLN2* (*Zm00001d029215*) shown to control PH in rice [171]. More intriguingly, in the syntenic region of rice MQTL-YLD15 in maize (ortho-MQTL-YLD15) the *OsSPL13* orthologous gene (*Zm00001d006451*) is located that shown to increase grain length and YLD in rice [135]. In addition, we identified three ortho-MQTLs for PH and YLD in both barley and maize (ortho-MQTL-PH6, ortho-MQTL-PH10, ortho-MQTL-YLD11), and their orthologous gene content are listed in Additional file 4. Further investigations are decisively recommended to explore the genetics mechanisms controlling these traits at these genomic intervals.

### Comparison of MQTLs with GWAS studies

Detected rice MQTLs were compared with GWAS studies in rice and barley led to identification of common significant loci that provides more confident MQTLs. Consequently, in rice 7 and 11 significant GWAS signals for GW and HD, respectively, were co-located with our MQTLs (Additional file 5). They were distributed on all chromosomes of rice except chromosomes 9, 11 and 12. These results indicate the compatibility and coherence of the two methods in identification of significant genomic regions corresponding to the studied traits. Four rice MQTLs including MQTL-GW4, MQTL-GW20, MQTL-GW24 and MQTL-HD4 on chromosome 1, 5, 7 and 2, respectively, were in co-linear with the syntenic regions containing significant GWAS signals for the same traits in barley [172, 173]. Five genes with a significant signal in barley GWAS had orthologous genes in the detected rice MQTLs intervals (Table 5), including *Vrs3* gene on chromosome 1H [172] that its orthologous in rice located at MQTL-GW20 on chromosome 5, a serine carboxypeptidase gene on barley 2H [173] and its orthologous in rice MQTL-GW24 on chromosome 7 that regulates GW [132]. However, this approach has limitations related to compatibility of the genome intervals between MQTLs and significant peaks in GWAS.

**Table 5:** Barley GWAS SNP-based association with rice MQTLs

Trait	Rice MQTLs	Rice chr. no. (rice gene ID)^a^	Barley chr. no. (The rice orthologous gene ID)	SNP BOPA marker name corresponding to the barley gene (genomic position in bp)	Barley gene annotation	Barley GWAS reference
GW	MQTL-GW4	1(*Os01g0837300*)	2H(*HORVU2Hr1G019180*)	11_0578 (49988534)	UDP-glucuronic acid decarboxylase 1	[172]
GW	MQTL-GW4	1(*Os01g0840200*)	3H(*HORVU3Hr1G086480*)	11_1113 (617339425)	HSP20-like chaperones superfamily protein	[172]
GW	MQTL-GW20	5(*Os05g0419800*)	1H(*HORVU1Hr1G064020*)	11_0004 (458109745)	GDSL esterase/lipase	[172]
GW	MQTL-GW24	7(*Os07g0479300*)	2H(*HORVU2Hr1G109380*)	11_0489 (716480871)	Carboxypeptidase Y	[173]
HD	MQTL-HD4	2(*Os02g0779200*)	6H(*HORVU6Hr1G081850*)	11_0701 (546608851)	Subtilisin-like protease	[172]

^a^The rice gene ID located at the corresponding MQTL interval with a barley orthologous gene containing a SNP with significant signal in GWAS analysis.

## Conclusions

In conclusion, we succeeded to define a genome wide landscape on the most stable loci that associate with genetic markers and CGs related to yield and yield-related traits in rice. Our findings show that MQTLs of evaluated important agronomic criteria appear at least partly to be transferable to other cereals and genome wide association studies that helps breeding programs in cereals.

## Methods

### Initial QTL studies used for MQTL analysis

A total of 1052 QTLs for YLD, HD, PH, GW and TN traits derived from 122 QTL populations resulted from cross between different combinations of 124 varieties under unstressed condition in rice were retrieved from 101 published studies since 1996 up to now. YLD was reported as grain yield per plant in 32 studies and as grain yield per hectare in 9 studies. Both 100 grain weight and 1000 grain weight were addressed as GW including 46 and 7 studies that reported 1000 and 100 grain weight, respectively. The size of the mapping populations varied from 77 to 1024 lines of various types including 13 DH, 27 F_2_, 33 BCs and 40 RIL populations evaluated on diverse locations and years (Table 1). The information on the QTLs consisting of traits, parents of the population, population type, map density and number of markers are summarized in Table 1.

### QTLs projection on reference map

The most comprehensive available genetic map integrated from six identified well-known genetic maps in rice, with the highest number and various types of markers [168], was selected as the reference map in the present study. This map is highly saturated with 6969 markers of 0.25 cM average distance that results in a total length of 1771.8 cM on 12 chromosomes with an average chromosome length of 147.65 cM.

The position, chromosome groups, the proportion of phenotype variance (R^2^), and the log of odds ratio (LOD score) were collected for each of the QTLs in the 122 used populations. In order to calculate 95% of CI for QTLs, we used the formula, CI=530/(N*R^2^) for BC and F_2_ lines, CI=287/(N*R^2^) for DH lines and CI=163/(N*R^2^) for RILs lines [174], where N is the population size and R^2^ is the proportion of phenotypic variance of the QTL. For QTLs without precisely defined LOD scores [39, 48, 77] and R^2^ [63, 69, 91], those criteria were arbitrarily quantified as 3 and 10%, respectively. All of the collected QTLs with proper information were projected onto the reference map by BioMercator V4.2 [12, 14]. In order to be able to incorporate QTLs derived from the studies based on SNP markers in our MQTL analysis, the position of the flanking markers were ascertained on the rice genome and closest markers on the reference map were used in our analysis. Consequently, 960 out of 1052 initial QTLs were successfully projected on the reference map.

### MQTL analysis

The meta-analysis was carried out for the integrated QTLs from different studies and relocated on consensus position of each MQTL using BioMercator V4.2 [12, 14]. The algorithms and statistical procedures implemented in this software are well-described in the literature [12, 14, 175]. The best model of estimated MQTLs was selected based on the prevalent value among AIC (Akaike information content), AICc (AIC correction), AIC3 (AIC 3 candidate models), BIC (Bayesian information criterion) and AWE (average weight of evidence) criteria and it was considered as the best fit. Consequently, the consensus QTL from the optimal model was reported as a MQTL. Mapchart V.2.32 software [176] was applied to demonstrate the MQTLs and related QTLs on the reference map. The position of MQTLs on the rice genome was shown as a heatmap using *pheatmap* and RIdeogram R package [177, 178]. To investigate the distribution of MQTLs towards centromeric and telomeric regions in the genomic point of view, the centromere position was retrieved from Cheng et al. (2002) and Kawahara et al. (2013) studies [179, 180] and shown on each chromosome. Moreover, to expand our genomic approaches, the distribution of MQTLs were compared to the selective sweep regions (domestication loci) and functional variants in coding regions with strong alteration in allele frequency between cultivated and wild rice [131]. Additionally, the distributions of gene density, QTLs and MQTLs were drawn on chromosomes using SOFIA R package [181]. Finally, all the detected rice MQTLs were compared with the position of significant loci related to the traits resulted from Genome Wide Association Studies (GWAS) using the Rice SNP-seek database [182].

### Identification of candidate genes

To determine CGs related to YLD, TN, GW, PH and HD traits located at the corresponding region of each detected MQTL, the rice genome (IRGSP-1.0) was investigated in EnsemblPlants (https://plants.ensembl.org/index.html) using the position of flanking markers obtained from the Gramene (http://archive.gramene.org/qtl/) database. For those flanking markers without genomic position, the closest markers from consensus genetic reference map were exploited to project the MQTL on the genome. Consequently, all the genes underlying the genomic region of each MQTL were functionally annotated by EnsemblPlants and FunRiceGenes (https://funricegenes.github.io/) [183], and CGs were introduced based on their description and putative function in rice and closely related species.

### Ortho-MQTL mining in barley and maize

Based on the high synteny among rice, barley and maize, the most promising rice MQTLs containing initial QTLs from at least three independent studies were explored for identification of ortho-MQTLs of the same traits in barley [17], and maize [23–26] MQTLs. The syntenic regions were identified based on investigation of a set of orthologous genes at each MQTL position using EnsemblPlants database.

Moreover, the most promising rice MQTLs were compared with the significant loci resulted from GWAS studies in barley. In barley, significant SNP signals of the orthologous genes located at the rice MQTL regions were surveyed in the following GWAS studies: Pasam et al. 2012; Tondelli et al. 2013; Locatelli et al. 2013; Pauli et al. 2014; Mora et al. 2016; Bellucci et al. 2017 [172, 173, 184–187]. The genomic position of barley genes were retrieved from BARLEX [188] and T3/Barley (https://triticeaetoolbox.org/barley/) databases.

## Supplementary information

**Additional file 1.** The chromosomal location of MQTLs and initial QTLs for YLD, HD, PH, GW and TN on 12 chromosomes of rice. MQTLs are shown on each chromosome and the lines on the right side of chromosomes indicate the CI of initial QTLs with 95% confidence intervals. Each color represents a specific trait; GW, HD, PH, YLD and TN are presented in red, dark green, blue, light green and purple, respectively. The markers are shown on the right side of chromosomes. The genetic distance (cM) is indicated on the left side of each chromosome.

**Additional file 2.** The list of CGs and all annotated genes anchoring at each MQTL interval. In the list of CGs, the highlighted genes in green indicate the proved cloned genes.

**Additional file 3.** The list of MQTLs located at selective sweep regions and functional variants on coding regions with well-known genes.

**Additional file 4.** The orthologous genes located at the syntenic regions of each ortho-MQTLs.

**Additional file 5.** The genomic position of MQTLs co-located at GWAS significant results related to GW and HD (Rice SNP-Seek Database) on all chromosomes of rice.

## Data Availability

The relevant data and additional information are available in the supplementary files and also from the corresponding author upon reasonable request.

## Abbreviations

AIC: Akaike information criterion
AICs: AIC correction
AIC3: AIC 3 candidate models
AWE: Average weight of evidence
BBX: B-box
BIC: Bayesian information criterion
CGs: Candidate Genes
bZIP: Basic region/leucine zipper motif
CI: Confidence Interval
GW: Grain Weight
HD: Heading Date
LOD: The log of odds ratio
MQTL: Meta-QTL
MAS: Marker-Assisted Selection
PH: Plant Height
QTL: Quantitative Trait loci
R^2^: The proportion of phenotype variance
TBL: Trichome Birefringence-like proteins
TN: Tiller Number
YLD: Yield

## References

[1] Sakamoto T, Matsuoka M (2008). Identifying and exploiting grain yield genes in rice. Curr Opin Plant Biol.

[2] Xing Y, Zhang Q (2010). Genetic and molecular bases of rice yield. Annu Rev Plant Biol.

[3] Marathi B, Guleria S, Mohapatra T, Parsad R, Mariappan N, Kurungara VK (2012). QTL analysis of novel genomic regions associated with yield and yield related traits in new plant type based recombinant inbred lines of rice (*Oryza sativa* L.). BMC Plant Biol.

[4] Surapaneni M, Balakrishnan D, Mesapogu S, Addanki KR, Yadavalli VR, Tripura Venkata V (2017). Identification of major effect QTLs for agronomic traits and CSSLs in rice from Swarna/*Oryza nivara* derived backcross inbred lines. Front Plant Sci.

[5] Sellamuthu R, Liu GF, Ranganathan CB, Serraj R (2011). Genetic analysis and validation of quantitative trait loci associated with reproductive-growth traits and grain yield under drought stress in a doubled haploid line population of rice (*Oryza sativa* L.). Field Crop Res.

[6] Brambilla V, Fornara F (2013). Molecular control of flowering in response to day length in rice. J J Integr Plant Biol.

[7] Hori K, Matsubara K, Yano M (2016). Genetic control of flowering time in rice: integration of Mendelian genetics and genomics. Theor Appl Genet.

[8] Bilgrami SS, Fakheri BA, Razavi K, Mahdinezhad N, Tavakol E, Ramandi HD, Ghaderian M, Shariati JV (2018). Evaluation of agro-morphological traits related to grain yield of Iranian wheat genotypes in drought-stress and normal irrigation conditions. Aust J Crop Sci.

[9] Bai X, Wu B, Xing Y (2012). Yield-related QTLs and Their Applications in Rice Genetic Improvement. J Integr Plant Biol.

[10] Daware AV, Srivastava R, Singh AK, Parida SK, Tyagi AK (2017). Regional association analysis of metaQTLs delineates candidate grain size genes in rice. Front Plant Sci.

[11] Zhang LY, Liu DC, Guo XL, Yang WL, Sun JZ, Wang DW (2010). Genomic distribution of quantitative trait loci for yield and yield-related traits in common wheat. J Integr Plant Biol.

[12] Arcade A, Labourdette A, Falque M, Mangin B, Chardon F, Charcosset A (2004). BioMercator: integrating genetic maps and QTL towards discovery of candidate genes. Bioinformatics.

[13] Goffinet B, Gerber S (2000). Quantitative trait loci: a meta-analysis. Genetics.

[14] Sosnowski O, Charcosset A, Joets J (2012). BioMercator V3: an upgrade of genetic map compilation and quantitative trait loci meta-analysis algorithms. Bioinformatics..

[15] Martinez AK, Soriano JM, Tuberosa R, Koumproglou R, Jahrmann T, Salvi S (2016). Yield QTLome distribution correlates with gene density in maize. Plant Sci.

[16] Zhang X, Shabala S, Koutoulis A, Shabala L, Zhou M (2017). Meta-analysis of major QTL for abiotic stress tolerance in barley and implications for barley breeding. Planta..

[17] Khahani B, Tavakol E, Shariati V (2019). Genome-wide meta-analysis on yield and yield-related QTLs in barley (*Hordeum vulgare* L.). Mol Breed.

[18] Quraishi UM, Abrouk M, Murat F, Pont C, Foucrier S, Desmaizieres G (2011). Cross-genome map based dissection of a nitrogen use efficiency ortho-metaQTL in bread wheat unravels concerted cereal genome evolution. Plant J.

[19] Acuña-Galindo MA, Mason RE, Subramanian NK, Hays DB (2015). Meta-analysis of wheat QTL regions associated with adaptation to drought and heat stress. Crop Sci.

[20] Darzi-Ramandi H, Shariati JV, Tavakol E, Najafi-Zarini H, Bilgrami SS, Razavi K (2017). Detection of consensus genomic regions associated with root architecture of bread wheat on groups 2 and 3 chromosomes using QTL meta-analysis. Aust J Crop Sci.

[21] Hwang S, King CA, Chen P, Ray JD, Cregan PB, Carter TE (2016). Meta-analysis to refine map position and reduce confidence intervals for delayed-canopy-wilting QTLs in soybean. Mol Breed.

[22] Qin H, Liu Z, Wang Y, Xu M, Mao X, Qi H (2018). Meta-analysis and overview analysis of quantitative trait locis associated with fatty acid content in soybean for candidate gene mining. Plant Breed.

[23] Semagn K, Beyene Y, Warburton ML, Tarekegne A, Mugo S, Meisel B (2013). Meta-analyses of QTL for grain yield and anthesis silking interval in 18 maize populations evaluated under water-stressed and well-watered environments. BMC Genomics.

[24] Wang Y, Huang Z, Deng D, Ding H, Zhang R, Wang S (2013). Meta-analysis combined with syntenic metaQTL mining dissects candidate loci for maize yield. Mol Breed.

[25] Wang Y, Xu J, Deng D, Ding H, Bian Y, Yin Z (2016). A comprehensive meta-analysis of plant morphology, yield, stay-green, and virus disease resistance QTL in maize (*Zea mays* L.). Planta.

[26] Chen L, An Y, Li Y-x, Li C, Shi Y, Song Y (2017). Candidate loci for yield-related traits in maize revealed by a combination of metaQTL analysis and regional association mapping. Front Plant Sci.

[27] Zhao X, Peng Y, Zhang J, Fang P, Wu B (2018). Identification of QTLs and meta-QTLs for seven agronomic traits in multiple maize populations under well-watered and water-stressed conditions. Crop Sci.

[28] Swamy BM, Sarla N (2011). Meta-analysis of yield QTLs derived from inter-specific crosses of rice reveals consensus regions and candidate genes. Plant Mol Biol Report.

[29] Lei L, Zheng H, Wang J, Liu H, Sun J, Zhao H (2018). Genetic dissection of rice (*Oryza sativa* L.) tiller, plant height, and grain yield based on QTL mapping and metaanalysis. Euphytica.

[30] Mayer KF, Martis M, Hedley PE, Šimková H, Liu H, Morris JA (2011). Unlocking the barley genome by chromosomal and comparative genomics. Plant Cell.

[31] Hirsch CN, Foerster JM, Johnson JM, Sekhon RS, Muttoni G, Vaillancourt B (2014). Insights into the maize pan-genome and pan-transcriptome. Plant Cell.

[32] Lin H-X, Qian H-R, Zhuang J-Y, Lu J, Min S-K, Xiong Z-M (1996). RFLP mapping of QTLs for yield and related characters in rice (*Oryza sativa* L.). Theor Appl Genet.

[33] Lu C-f, Shen L-s, Tan Z, Xu Y, He P, Chen Y (1996). Comparative mapping of QTLs for agronomic traits of rice across environments using a doubled haploid population. Theor Appl Genet.

[34] Wu P, Zhang G, Huang N (1996). Identification of QTLs controlling quantitative characters in rice using RFLP markers. Euphytica..

[35] Yano M, Harushima Y, Nagamura Y, Kurata N, Minobe Y, Sasaki T (1997). Identification of quantitative trait loci controlling heading date in rice using a high-density linkage map. Theor Appl Genet.

[36] Yu S, Li J, Xu C, Tan Y, Gao Y, Li X (1997). Importance of epistasis as the genetic basis of heterosis in an elite rice hybrid. Proc Natl Acad Sci.

[37] Zhuang J-Y, Lin H-X, Lu J, Qian H-R, Hittalmani S, Huang N (1997). Analysis of QTL× environment interaction for yield components and plant height in rice. Theor Appl Genet.

[38] Lin S, Sasaki T, Yano M (1998). Mapping quantitative trait loci controlling seed dormancy and heading date in rice, *Oryza sativa* L., using backcross inbred lines. Theor. Appl. Genet..

[39] Xiao J, Li J, Grandillo S, Ahn SN, Yuan L, Tanksley SD (1998). Identification of trait-improving quantitative trait loci alleles from a wild rice relative, *Oryza rufipogon*. Genetics..

[40] Yamamoto T, Lin H, Sasaki T, Yano M (2000). Identification of heading date quantitative trait locus Hd6 and characterization of its epistatic interactions with Hd2 in rice using advanced backcross progeny. Genetics..

[41] Lin H, Yamamoto T, Sasaki T, Yano M (2000). Characterization and detection of epistatic interactions of 3 QTLs, Hd1, Hd2, and Hd3, controlling heading date in rice using nearly isogenic lines. Theor Appl Genet.

[42] Li J, Yu S, Xu C, Tan Y, Gao Y, Li X (2000). Analyzing quantitative trait loci for yield using a vegetatively replicated F2 population from a cross between the parents of an elite rice hybrid. Theor Appl Genet.

[43] Bres-Patry C, Lorieux M, Clement G, Bangratz M, Ghesquière A (2001). Heredity and genetic mapping of domestication-related traits in a temperate japonica weedy rice. Theor Appl Genet.

[44] Gong J, Zheng X, Du B, Qian Q, Chen S, Zhu L (2001). Comparative study of QTLs for agronomic traits of rice (*Oriza sativa* L.) between salt stress and nonstress environment. Sci China Life Sci.

[45] He P, Li J, Zheng X, Shen L, Lu C, Chen Y (2001). Comparison of molecular linkage maps and agronomic trait loci between DH and RIL populations derived from the same rice cross. Crop Sci.

[46] Takeuchi Y, Hayasaka H, Chiba B, Tanaka I, Shimano T, Yamagishi M (2001). Mapping quantitative trait loci controlling cool-temperature tolerance at booting stage in temperate japonica rice. Breed Sci.

[47] Ishimaru K, Yano M, Aoki N, Ono K, Hirose T, Lin S (2001). Toward the mapping of physiological and agronomic characters on a rice function map: QTL analysis and comparison between QTLs and expressed sequence tags. Theor Appl Genet.

[48] Yamamoto T, Taguchi-Shiobara F, Ukai Y, Sasaki T, Yano M (2001). Mapping quantitative trait loci for days-to-heading, and culm, panicle and internode lengths in a BC1F3 population using an elite rice variety, Koshihikari, as the recurrent parent. Breed Sci.

[49] Lin H, Ashikari M, Yamanouchi U, Sasaki T, Yano M (2002). Identification and characterization of a quantitative trait locus, Hd9, controlling heading date in rice. Breed Sci.

[50] Brondani C, Rangel P, Brondani R, Ferreira M (2002). QTL mapping and introgression of yield-related traits from Oryza glumaepatula to cultivated rice (*Oryza sativa*) using microsatellite markers. Theor Appl Genet.

[51] Yoshida S, Ikegami M, Kuze J, Sawada K, Hashimoto Z, Ishii T (2002). QTL analysis for plant and grain characters of sake-brewing rice using a doubled haploid population. Breed Sci.

[52] Xing Y, Tan Y, Hua J, Sun X, Xu C, Zhang Q (2002). Characterization of the main effects, epistatic effects and their environmental interactions of QTLs on the genetic basis of yield traits in rice. Theor Appl Genet.

[53] Hua J, Xing Y, Xu C, Sun X, Yu S, Zhang Q (2002). Genetic dissection of an elite rice hybrid revealed that heterozygotes are not always advantageous for performance. Genetics..

[54] Zhuang J-Y, Fan Y-Y, Rao Z-M, Wu J-L, Xia Y-W, Zheng K-L (2002). Analysis on additive effects and additive-by-additive epistatic effects of QTLs for yield traits in a recombinant inbred line population of rice. Theor Appl Genet.

[55] Hittalmani S, Shashidhar H, Bagali PG, Huang N, Sidhu J, Singh V (2002). Molecular mapping of quantitative trait loci for plant growth, yield and yield related traits across three diverse locations in a doubled haploid rice population. Euphytica..

[56] Kennard W, Phillips R, Porter R (2002). Genetic dissection of seed shattering, agronomic, and color traits in American wildrice (*Zizania palustris* var. interior L.) with a comparative map. Theor. Appl. Genet..

[57] Septiningsih E, Prasetiyono J, Lubis E, Tai T, Tjubaryat T, Moeljopawiro S (2003). Identification of quantitative trait loci for yield and yield components in an advanced backcross population derived from the *Oryza sativa* variety IR64 and the wild relative *O. rufipogon*. Theor. Appl. Genet..

[58] Thomson M, Tai T, McClung A, Lai X, Hinga M, Lobos K (2003). Mapping quantitative trait loci for yield, yield components and morphological traits in an advanced backcross population between Oryza rufipogon and the *Oryza sativa* cultivar Jefferson. Theor Appl Genet.

[59] Courtois B, Shen L, Petalcorin W, Carandang S, Mauleon R, Li Z (2003). Locating QTLs controlling constitutive root traits in the rice population IAC 165× Co39. Euphytica..

[60] Mei H, Luo L, Ying C, Wang Y, Yu X, Guo L (2003). Gene actions of QTLs affecting several agronomic traits resolved in a recombinant inbred rice population and two testcross populations. Theor Appl Genet.

[61] Hittalmani S, Huang N, Courtois B, Venuprasad R, Shashidhar H, Zhuang J (2003). Identification of QTL for growth-and grain yield-related traits in rice across nine locations of Asia. Theor Appl Genet.

[62] Babu RC, Nguyen BD, Chamarerk V, Shanmugasundaram P, Chezhian P, Jeyaprakash P (2003). Genetic analysis of drought resistance in rice by molecular markers. Crop Sci.

[63] Hua J, Xing Y, Wu W, Xu C, Sun X, Yu S (2003). Single-locus heterotic effects and dominance by dominance interactions can adequately explain the genetic basis of heterosis in an elite rice hybrid. Proc Natl Acad Sci.

[64] Kobayashi S, Fukuta Y, Sato T, Osaki M, Khush G (2003). Molecular marker dissection of rice (*Oryza sativa* L.) plant architecture under temperate and tropical climates. Theor. Appl. Genet..

[65] Xu C, Li X, Xue Y, Huang Y, Gao J, Xing YZ (2004). Comparison of quantitative trait loci controlling seedling characteristics at two seedling stages using rice recombinant inbred lines. Theor Appl Genet.

[66] Lanceras JC, Pantuwan G, Jongdee B, Toojinda T (2004). Quantitative trait loci associated with drought tolerance at reproductive stage in rice. Plant Physiol.

[67] Abdelkhalik AF, Shishido R, Nomura K, Ikehashi H (2005). QTL-based analysis of heterosis for grain shape traits and seedling characteristics in an indica-japonica hybrid in rice (*Oryza sativa* L.). Breed Sci.

[68] Guo L, Xing YZ, Mei H, Xu C, Shi C, Wu P (2005). Dissection of component QTL expression in yield formation in rice. Plant Breed.

[69] Zou G, Mei H, Liu H, Liu G, Hu S, Yu X (2005). Grain yield responses to moisture regimes in a rice population: association among traits and genetic markers. Theor Appl Genet.

[70] Mei H, Li Z, Shu Q, Guo L, Wang Y, Yu X (2005). Gene actions of QTLs affecting several agronomic traits resolved in a recombinant inbred rice population and two backcross populations. Theor Appl Genet.

[71] Marri PR, Sarla N, Reddy LV, Siddiq E (2005). Identification and mapping of yield and yield related QTLs from an Indian accession of Oryza rufipogon. BMC Genet.

[72] Nakagawa H, Yamagishi J, Miyamoto N, Motoyama M, Yano M, Nemoto K (2005). Flowering response of rice to photoperiod and temperature: a QTL analysis using a phenological model. Theor Appl Genet.

[73] You A, Lu X, Jin H, Ren X, Liu K, Yang G (2006). Identification of quantitative trait loci across recombinant inbred lines and testcross populations for traits of agronomic importance in rice. Genetics..

[74] Wada T, Uchimura Y, Ogata T, Tsubone M, Matsue Y (2006). Mapping of QTLs for physicochemical properties in japonica rice. Breed Sci.

[75] Manickavelu A, Nadarajan N, Ganesh S, Gnanamalar R, Babu RC (2006). Drought tolerance in rice: morphological and molecular genetic consideration. Plant Growth Regul.

[76] Zhang Y, Luo L, Xu C, Zhang Q, Xing Y (2006). Quantitative trait loci for panicle size, heading date and plant height co-segregating in trait-performance derived near-isogenic lines of rice (*Oryza sativa*). Theor Appl Genet.

[77] Tian F, Li DJ, Fu Q, Zhu ZF, Fu YC, Wang XK (2006). Construction of introgression lines carrying wild rice (*Oryza rufipogon* Griff.) segments in cultivated rice (*Oryza sativa* L.) background and characterization of introgressed segments associated with yield-related traits. Theor. Appl. Genet..

[78] Li C, Zhou A, Sang T (2006). Genetic analysis of rice domestication syndrome with the wild annual species. Oryza nivara New Phytol.

[79] Li S-B, Zhang Z-H, Hu Y, Li C-Y, Jiang X, Mao T (2006). Genetic dissection of developmental behavior of crop growth rate and its relationships with yield and yield related traits in rice. Plant Sci.

[80] Yoo J-H, Yoo S-C, Zhang H, Cho S-H, Paek N-C (2007). Identification of QTL for early heading date of H143 in rice. J Crop Sci Biotechnol.

[81] Uga Y, Nonoue Y, Liang Z, Lin H, Yamamoto S, Yamanouchi U (2007). Accumulation of additive effects generates a strong photoperiod sensitivity in the extremely late-heading rice cultivar ‘Nona Bokra’. Theor Appl Genet.

[82] Yan C-J, Zhou J-H, Yan S, Chen F, Yeboah M, Tang S-Z (2007). Identification and characterization of a major QTL responsible for erect panicle trait in japonica rice (*Oryza sativa* L.). Theor. Appl. Genet..

[83] Bernier J, Kumar A, Ramaiah V, Spaner D, Atlin G (2007). A large-effect QTL for grain yield under reproductive-stage drought stress in upland rice. Crop Sci.

[84] Cho Y-G, Kang H-J, Lee J-S, Lee Y-T, Lim S-J, Gauch H (2007). Identification of quantitative trait loci in rice for yield, yield components, and agronomic traits across years and locations. Crop Sci.

[85] Rahman M, Chu SH, Choi M-S, Qiao YL, Jiang W, Piao R, et al. Identification of QTLs for some agronomic traits in rice using an introgression line from *Oryza minuta*. Mol Cells. 2007;24(1):16–26. 17846495

[86] Nonoue Y, Fujino K, Hirayama Y, Yamanouchi U, Lin S, Yano M (2008). Detection of quantitative trait loci controlling extremely early heading in rice. Theor Appl Genet.

[87] Srinivasan S, Gomez SM, Kumar SS, Ganesh S, Biji K, Senthil A (2008). QTLs linked to leaf epicuticular wax, physio-morphological and plant production traits under drought stress in rice (*Oryza sativa* L.). Plant Growth Regul.

[88] Matsubara K, Kono I, Hori K, Nonoue Y, Ono N, Shomura A (2008). Novel QTLs for photoperiodic flowering revealed by using reciprocal backcross inbred lines from crosses between japonica rice cultivars. Theor Appl Genet.

[89] Kwon S-J, Cho Y-C, Kwon S-W, Oh C-S, Suh J-P, Shin Y-S (2008). QTL mapping of agronomic traits using an RIL population derived from a cross between temperate japonica cultivars in rice (*Oryza sativa* L.). Breed Sci.

[90] Ma L, Yang C, Zeng D, Cai J, Li X, Ji Z (2009). Mapping QTLs for heading synchrony in a doubled haploid population of rice in two environments. J Genet Genomics.

[91] Subashri M, Robin S, Vinod K, Rajeswari S, Mohanasundaram K, Raveendran T (2009). Trait identification and QTL validation for reproductive stage drought resistance in rice using selective genotyping of near flowering RILs. Euphytica..

[92] Gomez SM, Boopathi NM, Kumar SS, Ramasubramanian T, Chengsong Z, Jeyaprakash P (2010). Molecular mapping and location of QTLs for drought-resistance traits in indica rice (*Oryza sativa* L.) lines adapted to target environments. Acta Physiol Plant.

[93] Thanh PT, Phan PDT, Ishikawa R, Ishii T (2010). QTL analysis for flowering time using backcross population between *Oryza sativa* Nipponbare and *O. rufipogon*. Genes Genet Syst.

[94] Lin Y-R, Wu S-C, Chen S-E, Tseng T-H, Chen C-S, Kuo S-C (2011). Mapping of quantitative trait loci for plant height and heading date in two inter-subspecific crosses of rice and comparison across *Oryza genus*. Bot Stud.

[95] Liu T, Zhang Y, Zhang H, Xing Y (2011). Quantitative trait loci for the number of grains per panicle dependent on or independent of heading date in rice (*Oryza sativa* L.). Breed Sci.

[96] Bai XF, Luo LJ, Yan WH, Kovi MR, Xing YZ (2011). Quantitative trait loci for rice yield-related traits using recombinant inbred lines derived from two diverse cultivars. J Genet.

[97] Wang L, Wang A, Huang X, Zhao Q, Dong G, Qian Q (2011). Mapping 49 quantitative trait loci at high resolution through sequencing-based genotyping of rice recombinant inbred lines. Theor Appl Genet.

[98] Yu H, Xie W, Wang J, Xing Y, Xu C, Li X (2011). Gains in QTL detection using an ultra-high density SNP map based on population sequencing relative to traditional RFLP/SSR markers. PLoS One.

[99] Wang P, Zhou G, Cui K, Li Z, Yu S (2012). Clustered QTL for source leaf size and yield traits in rice (*Oryza sativa* L.). Mol Breed.

[100] Liang Y, Zhan X, Gao Z, Lin Z, Yang Z, Zhang Y (2012). Mapping of QTLs associated with important agronomic traits using three populations derived from a super hybrid rice Xieyou9308. Euphytica..

[101] Yun W, Jinping Z, Yong S, Jauhar A, Jianlong X, Zhikang L (2012). Identification of genetic overlaps for salt and drought tolerance using simple sequence repeat markers on an advanced backcross population in rice. Crop Sci.

[102] Sun L, Ma D, Yu H, Zhou F, Li Y, Luo L (2013). Identification of quantitative trait loci for grain size and the contributions of major grain-size QTLs to grain weight in rice. Mol Breed.

[103] Wang Y, Zang J, Sun Y, Ali J, Xu J, Li Z (2013). Background-independent quantitative trait loci for drought tolerance identified using advanced backcross introgression lines in rice. Crop Sci.

[104] Zhou S, Zhu M, Wang F, Huang J, Wang G (2013). Mapping of QTLs for yield and its components in a rice recombinant inbred line population. Pak J Bot.

[105] Wang H, Xu X, Zhan X, Zhai R, Wu W, Shen X (2013). Identification of qRL7, a major quantitative trait locus associated with rice root length in hydroponic conditions. Breed Sci.

[106] Duan M, Sun Z, Shu L, Tan Y, Yu D, Sun X (2013). Genetic analysis of an elite super-hybrid rice parent using high-density SNP markers. Rice..

[107] Xing W, Zhao H, Mei D (2014). Detection of main-effect and epistatic QTL for yield-related traits in rice under drought stress and normal conditions. Can J Plant Sci.

[108] Lee S, Jia MH, Jia Y, Liu G (2014). Tagging quantitative trait loci for heading date and plant height in important breeding parents of rice (*Oryza sativa*). Euphytica..

[109] Dixit S, Singh A, Cruz MTS, Maturan PT, Amante M, Kumar A (2014). Multiple major QTL lead to stable yield performance of rice cultivars across varying drought intensities. BMC Genet.

[110] Zhan X, Sun B, Lin Z, Gao Z, Yu P, Liu Q (2015). Genetic mapping of a QTL controlling source–sink size and heading date in rice. Gene..

[111] Subudhi PK, De Leon T, Singh PK, Parco A, Cohn MA, Sasaki T (2015). A chromosome segment substitution library of weedy rice for genetic dissection of complex agronomic and domestication traits. PLoS One.

[112] Xu F, Sun X, Chen Y, Huang Y, Tong C, Bao J (2015). Rapid identification of major QTLs associated with rice grain weight and their utilization. PLoS One.

[113] Ding Z, Lin Z, Li Q, Wu H, Xiang C, Wang J (2015). DNL1, encodes cellulose synthase-like D4, is a major QTL for plant height and leaf width in rice (*Oryza sativa* L.). Biochem. Biophys. Res. Commun..

[114] Zhang B, Ye W, Ren D, Tian P, Peng Y, Gao Y (2015). Genetic analysis of flag leaf size and candidate genes determination of a major QTL for flag leaf width in rice. Rice..

[115] F-y GAO, ZENG (2016). L-h, Ling Q, LU X-j, REN J-s, WU X-t, et al. QTL mapping of grain appearance quality traits and grain weight using a recombinant inbred population in rice (*Oryza sativa* L.). J Integr Agric.

[116] Tagle AG, Fujita D, Ebron LA, Telebanco-Yanoria MJ, Sasaki K, Ishimaru T (2016). Characterization of QTL for unique agronomic traits of new-plant-type rice varieties using introgression lines of IR64. Crop J.

[117] Khan MSK, Saeed M, Iqbal J (2016). Quantitative trait locus mapping for salt tolerance at maturity stage in indica rice using replicated F 2 population. Braz J Bot.

[118] Ma X, Fu Y, Zhao X, Jiang L, Zhu Z, Gu P (2016). Genomic structure analysis of a set of Oryza nivara introgression lines and identification of yield-associated QTLs using whole-genome resequencing. Sci Rep.

[119] Matsubara K, Yamamoto E, Kobayashi N, Ishii T, Tanaka J, Tsunematsu H (2016). Improvement of rice biomass yield through QTL-based selection. PLoS One.

[120] Zhao D, Li P, Wang L, Sun L, Xia D, Luo L (2017). Genetic dissection of large grain shape in rice cultivar ‘Nanyangzhan’and validation of a grain thickness QTL (qGT3. 1) and a grain length QTL (qGL3. 4). Mol Breed.

[121] Zhang S, He X, Zhao J, Cheng Y, Xie Z, Chen Y (2017). Identification and validation of a novel major QTL for harvest index in rice (*Oryza sativa* L.). Rice.

[122] Ogiso-Tanaka E, Tanaka T, Tanaka K, Nonoue Y, Sasaki T, Fushimi E, et al. Detection of novel QTLs qDTH4. 5 and qDTH6. 3, which confer late heading under short-day conditions, by SSR marker-based and QTL-seq analysis. Breed Sci. 2017;67(2):101–9. 10.1270/jsbbs.16096 PMC5445965 28588386

[123] Zhu M, Liu D, Liu W, Li D, Liao Y, Li J (2017). QTL mapping using an ultra-high-density SNP map reveals a major locus for grain yield in an elite rice restorer R998. Sci Rep.

[124] Solis J, Gutierrez A, Mangu V, Sanchez E, Bedre R, Linscombe S (2018). Genetic mapping of quantitative trait loci for grain yield under drought in rice under controlled greenhouse conditions. Front Chem.

[125] Bhattarai U, Subudhi PK (2018). Identification of drought responsive QTLs during vegetative growth stage of rice using a saturated GBS-based SNP linkage map. Euphytica..

[126] Bhatia D, Wing RA, Yu Y, Chougule K, Kudrna D, Lee S (2018). Genotyping by sequencing of rice interspecific backcross inbred lines identifies QTLs for grain weight and grain length. Euphytica..

[127] Jing L, Rui X, Chunchao W, Lan Q, Xiaoming Z, Wensheng W (2018). A heading date QTL, qHD7. 2, from wild rice (*Oryza rufipogon*) delays flowering and shortens panicle length under long-day conditions. Sci Rep.

[128] Subudhi PK, De Leon TB, Tapia R, Chai C, Karan R, Ontoy J (2018). Genetic interaction involving photoperiod-responsive Hd 1 promotes early flowering under long-day conditions in rice. Sci Rep.

[129] Xu Y, Zhang H, Hu J, Wang X, Huang M, Wang H (2018). Further QTL mapping for yield component traits using introgression lines in rice (*Oryza sativa* L.) under drought field environments. Euphytica.

[130] Swamy BM, Vikram P, Dixit S, Ahmed H, Kumar A (2011). Meta-analysis of grain yield QTL identified during agricultural drought in grasses showed consensus. BMC Genomics.

[131] Huang X, Kurata N, Wang Z-X, Wang A, Zhao Q, Zhao Y (2012). A map of rice genome variation reveals the origin of cultivated rice. Nature.

[132] Huang R, Jiang L, Zheng J, Wang T, Wang H, Huang Y (2013). Genetic bases of rice grain shape: so many genes, so little known. Trends Plant Sci.

[133] Schmidt R, Schippers JH, Mieulet D, Watanabe M, Hoefgen R, Guiderdoni E (2014). SALT-RESPONSIVE ERF1 is a negative regulator of grain filling and gibberellin-mediated seedling establishment in rice. Mol Plant.

[134] Hu J, Wang Y, Fang Y, Zeng L, Xu J, Yu H (2015). A rare allele of *GS2* enhances grain size and grain yield in rice. Mol Plant.

[135] Si L, Chen J, Huang X, Gong H, Luo J, Hou Q (2016). *OsSPL13* controls grain size in cultivated rice. Nat Genet.

[136] Yuan H, Fan S, Huang J, Zhan S, Wang S, Gao P (2017). *08SG2/OsBAK1* regulates grain size and number, and functions differently in Indica and Japonica backgrounds in rice. Rice..

[137] Heng Y, Wu C, Long Y, Luo S, Ma J, Chen J, Liu J, Zhang H, Ren Y, Wang M, Tan J (2018). *OsALMT7* maintains panicle size and grain yield in rice by mediating malate transport. Plant Cell.

[138] Na JK, Seo MH, Yoon IS, Lee YH, Lee KO, Kim DY (2012). Involvement of rice Polycomb protein *OsFIE2* in plant growth and seed size. Plant Biotechnol Rep.

[139] Zhao S, Zhao L, Liu F, Wu Y, Zhu Z, Sun C, Tan L (2016). *NARROW AND ROLLED LEAF 2* regulates leaf shape, male fertility, and seed size in rice. J Integr Plant Biol.

[140] Lee JH, Park SH, Ahn JH (2012). Functional conservation and diversification between rice *OsMADS22*/*OsMADS55* and Arabidopsis SVP proteins. Plant Sci.

[141] Duan Y, Li S, Chen Z, Zheng L, Diao Z, Zhou Y (2012). Dwarf and deformed flower 1, encoding an F-box protein, is critical for vegetative and floral development in rice (*Oryza sativa* L.). Plant J.

[142] Sun X, Zhang Z, Wu J, Cui X, Feng D, Wang K (2016). The Oryza sativa regulator *HDR1* associates with the kinase *OsK4* to control photoperiodic flowering. PLoS Genet.

[143] Brambilla V, Martignago D, Goretti D, Cerise M, Somssich M, de Rosa M (2017). Antagonistic transcription factor complexes modulate the floral transition in Rice. Plant Cell.

[144] Zhu S, Wang J, Cai M, Zhang H, Wu F, Xu Y (2017). The *OsHAPL1*-*DTH8*-*Hd1* complex functions as the transcription regulator to repress heading date in rice. J Exp Bot.

[145] Wuriyanghan H, Zhang B, Cao WH, Ma B, Lei G, Liu YF, Wei W, Wu HJ, Chen LJ, Chen HW, Cao YR (2009). The ethylene receptor *ETR2* delays floral transition and affects starch accumulation in rice. Plant Cell.

[146] Liu K, Yu Y, Dong A, Shen WH (2017). *SET DOMAIN GROUP701* encodes a H3K4-methytransferase and regulates multiple key processes of rice plant development. New Phytol.

[147] Gangappa SN, Botto JF (2014). The BBX family of plant transcription factors. Trends Plant Sci.

[148] Jain M, Nijhawan A, Arora R, Agarwal P, Ray S, Sharma P (2007). F-box proteins in rice. Genome-wide analysis, classification, temporal and spatial gene expression during panicle and seed development, and regulation by light and abiotic stress. Plant Physiol.

[149] Tsuji H (2011). Taoka K-i, Shimamoto K. Regulation of flowering in rice: two florigen genes, a complex gene network, and natural variation. Curr Opin Plant Biol.

[150] Huang C-K, Sie Y-S, Chen Y-F, Huang T-S, Lu C-A (2016). Two highly similar DEAD box proteins, *OsRH2* and *OsRH34*, homologous to eukaryotic initiation factor 4AIII, play roles of the exon junction complex in regulating growth and development in rice. BMC Plant Biol.

[151] Yano K, Ookawa T, Aya K, Ochiai Y, Hirasawa T, Ebitani T (2015). Isolation of a novel lodging resistance QTL gene involved in strigolactone signaling and its pyramiding with a QTL gene involved in another mechanism. Mol Plant.

[152] Tamiru M, Undan JR, Takagi H, Abe A, Yoshida K, Undan JQ (2015). A cytochrome P450, OsDSS1, is involved in growth and drought stress responses in rice (*Oryza sativa* L.). Plant Mol. Biol..

[153] Han Y, Jiang J, Liu H, Ma Q, Xu W, Xu Y (2005). Overexpression of *OsSIN*, encoding a novel small protein, causes short internodes in Oryza sativa. Plant Sci.

[154] Zhou Y, Tao Y, Zhu J, Miao J, Liu J, Liu Y (2017). GNS4, a novel allele of *DWARF11*, regulates grain number and grain size in a high-yield rice variety. Rice..

[155] Liu X, Feng Z, Zhou C, Ren Y, Mou C, Wu T (2016). Brassinosteroid (BR) biosynthetic gene lhdd10 controls late heading and plant height in rice (*Oryza sativa* L.). Plant Cell Rep.

[156] Lv Y, Shao G, Qiu J, Jiao G, Sheng Z, Xie L, Wu Y, Tang S, Wei X, Hu P (2017). *White Leaf and Panicle 2*, encoding a PEP-associated protein, is required for chloroplast biogenesis under heat stress in rice. J Exp Bot.

[157] Yang C, Ma Y, Li J (2016). The rice YABBY4 gene regulates plant growth and development through modulating the gibberellin pathway. J Exp Bot.

[158] Gao Y, He C, Zhang D, Liu X, Xu Z, Tian Y (2017). Two trichome birefringence-like proteins mediate xylan acetylation, which is essential for leaf blight resistance in rice. Plant Physiol.

[159] Wei X, Jiao G, Lin H, Sheng Z, Shao G, Xie L (2017). *GRAIN INCOMPLETE FILLING 2* regulates grain filling and starch synthesis during rice caryopsis development. J Integr Plant Biol.

[160] Zou X, Qin Z, Zhang C, Liu B, Liu J, Zhang C (2015). Over-expression of an S-domain receptor-like kinase extracellular domain improves panicle architecture and grain yield in rice. J Exp Bot.

[161] Tsukahara K, Sawada H, Kohno Y, Matsuura T, Mori IC, Terao T (2015). Ozone-induced rice grain yield loss is triggered via a change in panicle morphology that is controlled by *ABERRANT PANICLE ORGANIZATION 1* gene. PLoS One.

[162] Yang J, Luo D, Yang B, Frommer WB, Eom JS (2018). SWEET 11 and 15 as key players in seed filling in rice. New Phytol.

[163] Hussien A, Tavakol E, Horner DS, Muñoz-Amatriaín M, Muehlbauer GJ, Rossini L. Genetics of tillering in rice and barley. Plant Genome. 2014;7(1):1–20.

[164] Tavakol E, Okagaki R, Verderio G, Shariati V, Hussien A, Bilgic H (2015). The barley *Uniculme4* gene encodes a BLADE-ON-PETIOLE-like protein that controls tillering and leaf patterning. Plant Physiol.

[165] Jung H, Lee D-K, Do Choi Y, Kim J-K (2015). *OsIAA6*, a member of the rice Aux/IAA gene family, is involved in drought tolerance and tiller outgrowth. Plant Sci.

[166] Zhao L, Tan L, Zhu Z, Xiao L, Xie D, Sun C (2015). *PAY* 1 improves plant architecture and enhances grain yield in rice. Plant J.

[167] Lu G, Coneva V, Casaretto JA, Ying S, Mahmood K, Liu F (2015). *OsPIN5b* modulates rice (*Oryza sativa*) plant architecture and yield by changing auxin homeostasis, transport and distribution. Plant J.

[168] Wu Y, Huang M, Tao X, Guo T, Chen Z, Xiao W (2016). Quantitative trait loci identification and meta-analysis for rice panicle-related traits. Mol Gen Genomics.

[169] Tavakol E, Elbadry N, Tondelli A, Cattivelli L, Rossini L (2016). Genetic dissection of heading date and yield under Mediterranean dry climate in barley (*Hordeum vulgare* L.). Euphytica..

[170] Gaut BS (2002). Evolutionary dynamics of grass genomes. New Phytol.

[171] Wu S, Xie Y, Zhang J, Ren Y, Zhang X, Wang J (2015). *VLN2* regulates plant architecture by affecting microfilament dynamics and polar auxin transport in rice. Plant Cell.

[172] Pasam RK, Sharma R, Malosetti M, van Eeuwijk FA, Haseneyer G, Kilian B (2012). Genome-wide association studies for agronomical traits in a world wide spring barley collection. BMC Plant Biol.

[173] Locatelli A, Cuesta-Marcos A, Gutiérrez L, Hayes PM, Smith KP, Castro AJ (2013). Genome-wide association mapping of agronomic traits in relevant barley germplasm in Uruguay. Mol Breed.

[174] Darvasi A, Soller M (1997). A simple method to calculate resolving power and confidence interval of QTL map location. Behav Genet.

[175] Veyrieras JB, Goffinet B, Charcosset A (2007). MetaQTL: a package of new computational methods for the meta-analysis of QTL mapping experiments. BMC bioinformatics.

[176] Voorrips R (2002). MapChart: software for the graphical presentation of linkage maps and QTLs. J.Hered..

[177] Kolde R. pheatmap: Pretty Heatmaps. R package version 0.7. 7. 2013.

[178] Hao Z, Lv D, Ge Y, Shi J, Weijers D, Yu G, Chen J (2020). RIdeogram: drawing SVG graphics to visualize and map genome-wide data on the idiograms. PeerJ Comput Sci.

[179] Cheng Z, Dong F, Langdon T, Ouyang S, Buell CR, Gu M (2002). Functional rice centromeres are marked by a satellite repeat and a centromere-specific retrotransposon. Plant Cell.

[180] Kawahara Y, de la Bastide M, Hamilton JP, Kanamori H, McCombie WR, Ouyang S (2013). Improvement of the *Oryza sativa* Nipponbare reference genome using next generation sequence and optical map data. Rice..

[181] Diaz-Garcia L, Covarrubias-Pazaran G, Schlautman B, Zalapa J (2017). SOFIA: an R package for enhancing genetic visualization with Circos. J Hered.

[182] Mansueto L, Fuentes RR, Borja FN, Detras J, Abriol-Santos JM, Chebotarov D (2016). Rice SNP-seek database update: new SNPs, indels, and queries. Nucleic Acids Res.

[183] Yao W, Li G, Yu Y, Ouyang Y (2017). FunRiceGenes dataset for comprehensive understanding and application of rice functional genes. Gigascience.

[184] Tondelli A, Xu X, Moragues M, Sharma R, Schnaithmann F, Ingvardsen C, et al. Structural and temporal variation in genetic diversity of European spring two-row barley cultivars and association mapping of quantitative traits. Plant Genome. 2013;6(2):1–14.

[185] Pauli D, Muehlbauer GJ, Smith KP, Cooper B, Hole D, Obert DE, et al. Association mapping of agronomic QTLs in US spring barley breeding germplasm. Plant Genome. 2014;7(3):1–15.

[186] Mora F, Quitral YA, Matus I, Russell J, Waugh R, Del Pozo A (2016). SNP-based QTL mapping of 15 complex traits in barley under rain-fed and well-watered conditions by a mixed modeling approach. Front Plant Sci.

[187] Bellucci A, Tondelli A, Fangel JU, Torp AM, Xu X, Willats WG (2017). Genome-wide association mapping in winter barley for grain yield and culm cell wall polymer content using the high-throughput CoMPP technique. PLoS One.

[188] Mascher M, Gundlach H, Himmelbach A, Beier S, Twardziok SO, Wicker T (2017). A chromosome conformation capture ordered sequence of the barley genome. Nature..

