# Supplementary material for: Genome wide screening and comparative genome analysis for Meta-QTLs, ortho-MQTLs and candidate genes controlling yield and yield-related traits in rice
Source: BMC Genomics. 2020 Apr 10;21:294. doi: 10.1186/s12864-020-6702-1 (PMC7146888; doi:10.1186/s12864-020-6702-1)
Supplement: Supplementary file 1 — Additional file 1. The chromosomal location of MQTLs and initial QTLs for YLD, HD, PH, GW and TN on 12 chromosomes of rice. MQTLs are shown on each chromosome and the lines on the right side of chromosomes indicate the CI of initial QTLs with 95% confidence intervals. Each color represents a specific trait; GW, HD, PH, YLD and TN are presented in red, dark green, blue, light green and purple, respectively. The markers are shown on the right side of chromosomes. The genetic distance (cM) is indicated on the left side of each chromosome. [file 12864_2020_6702_MOESM1_ESM.docx]

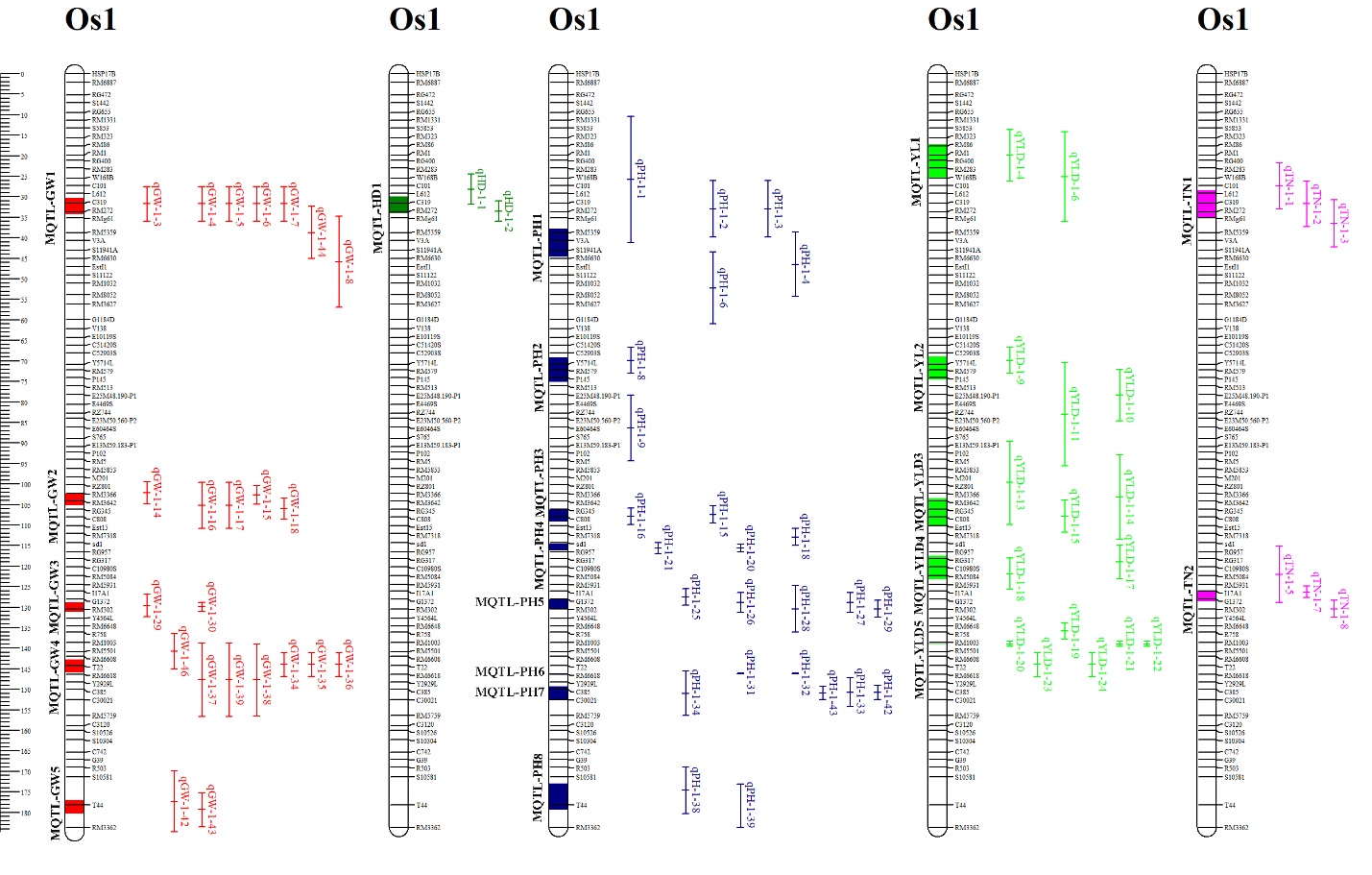

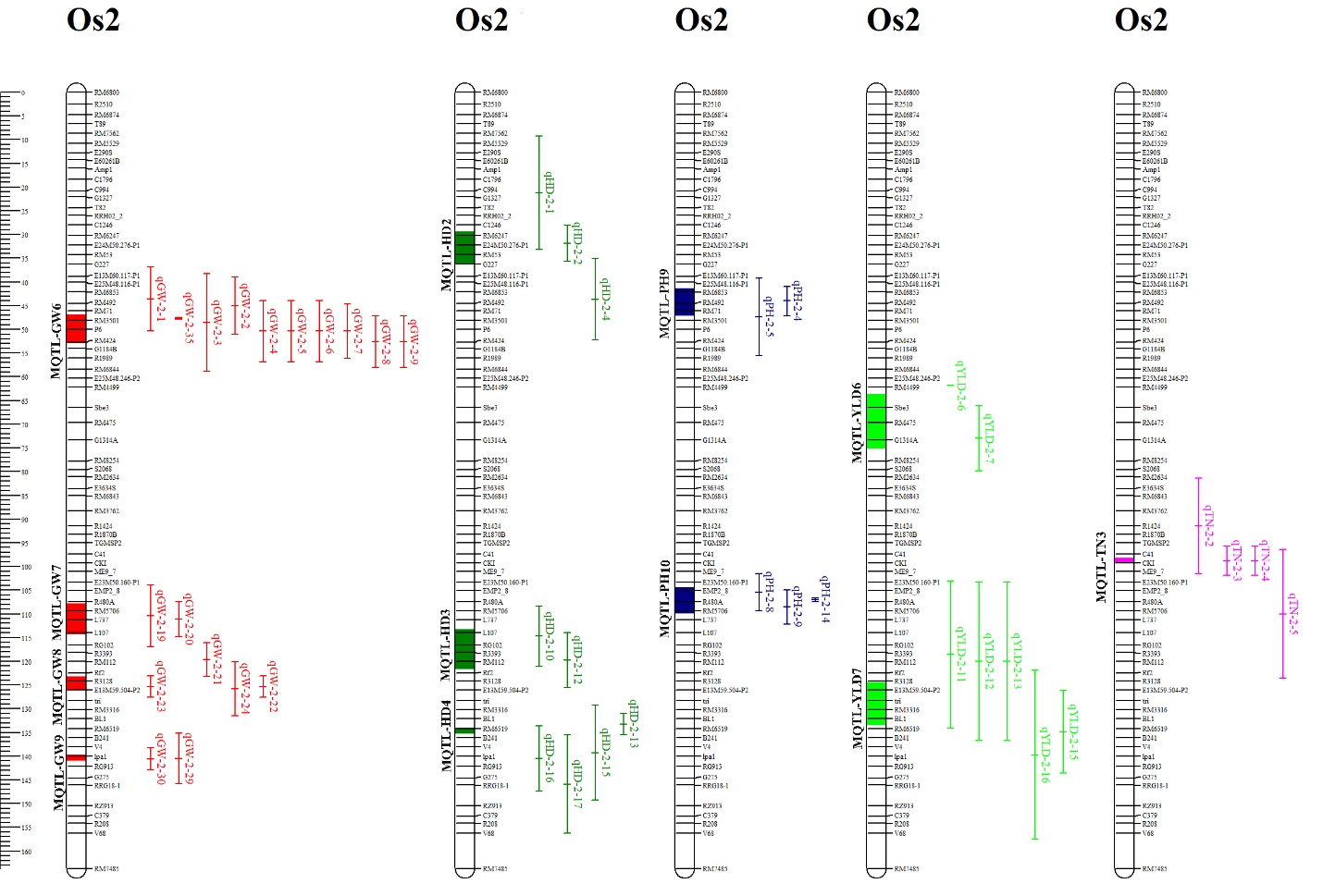


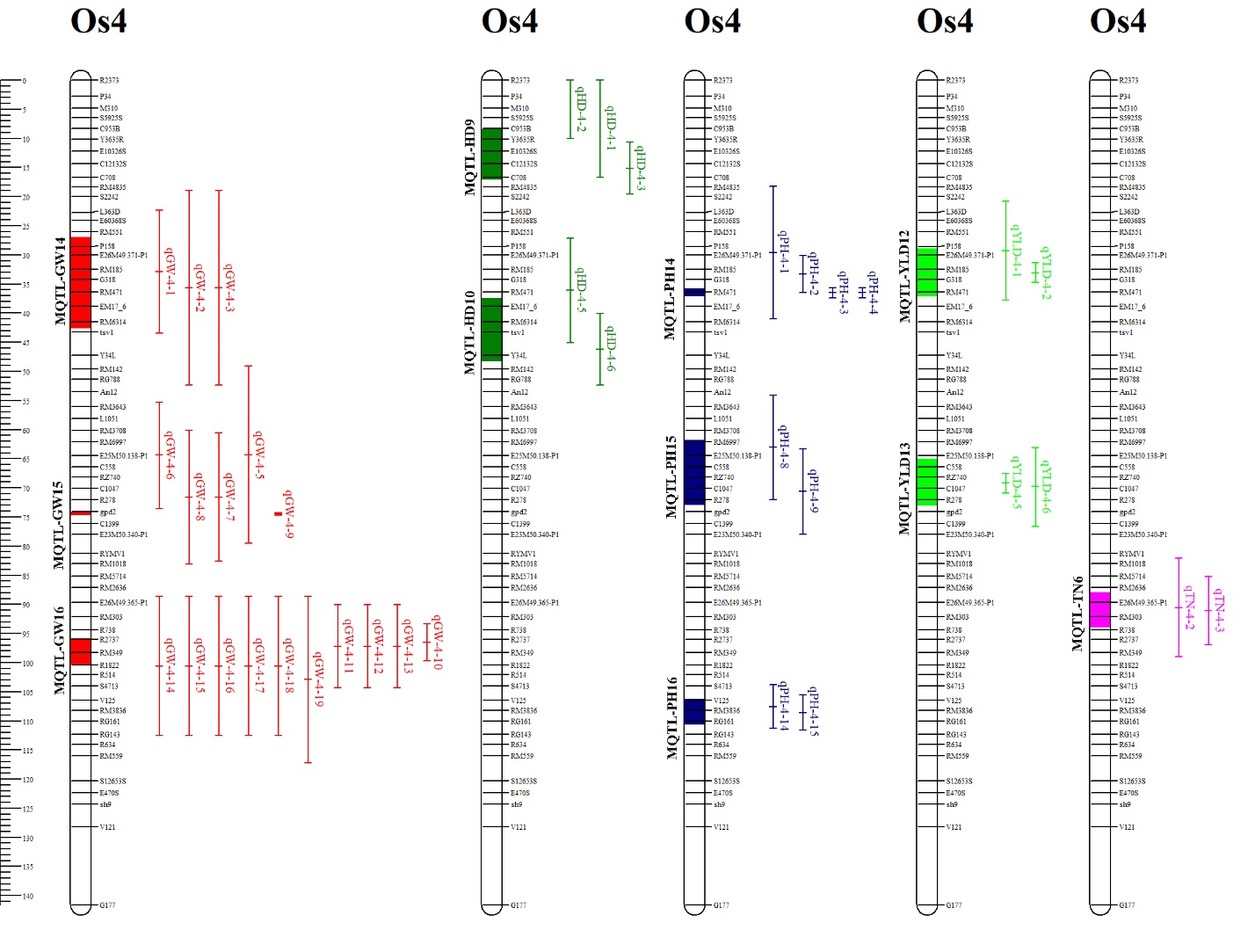

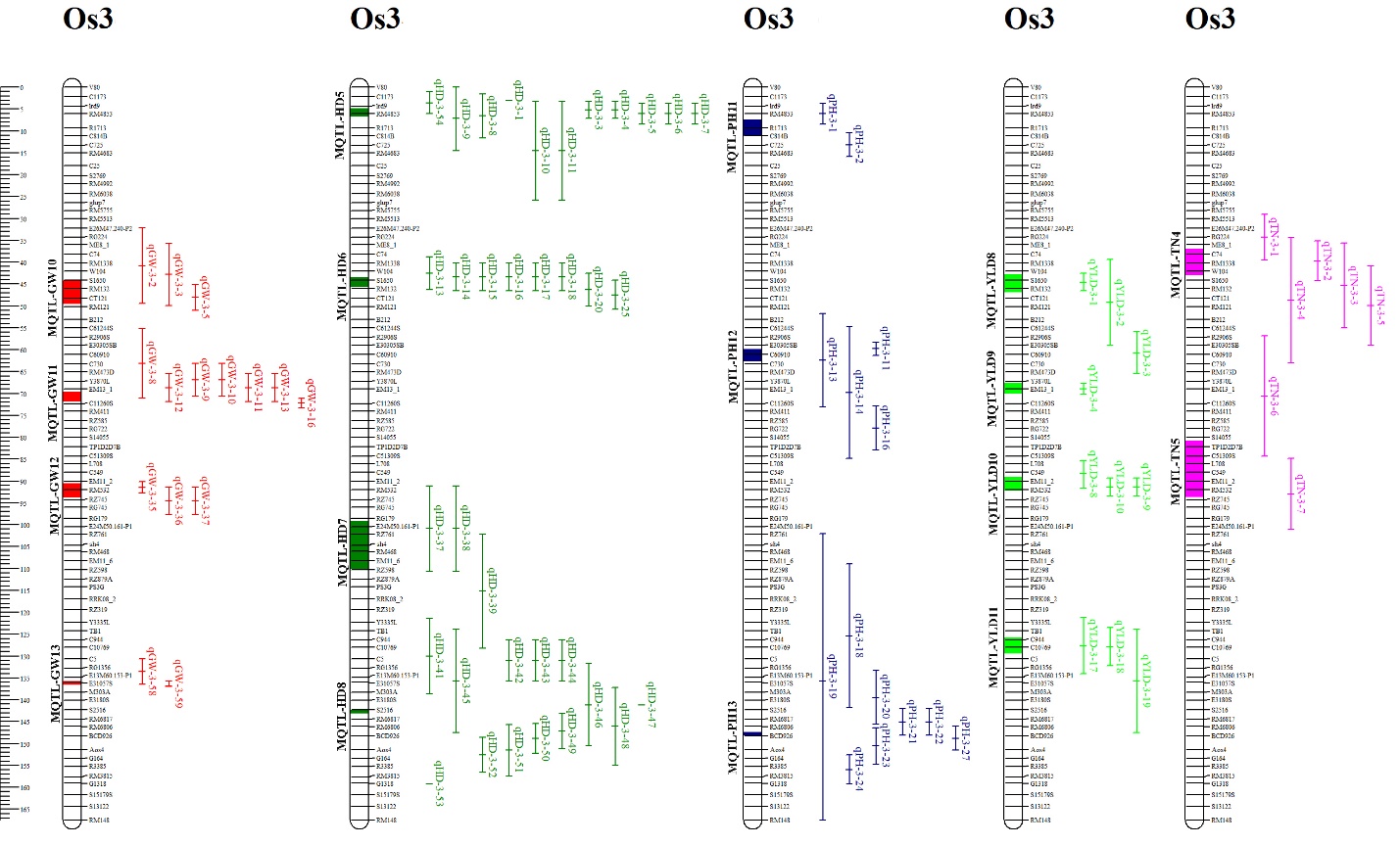


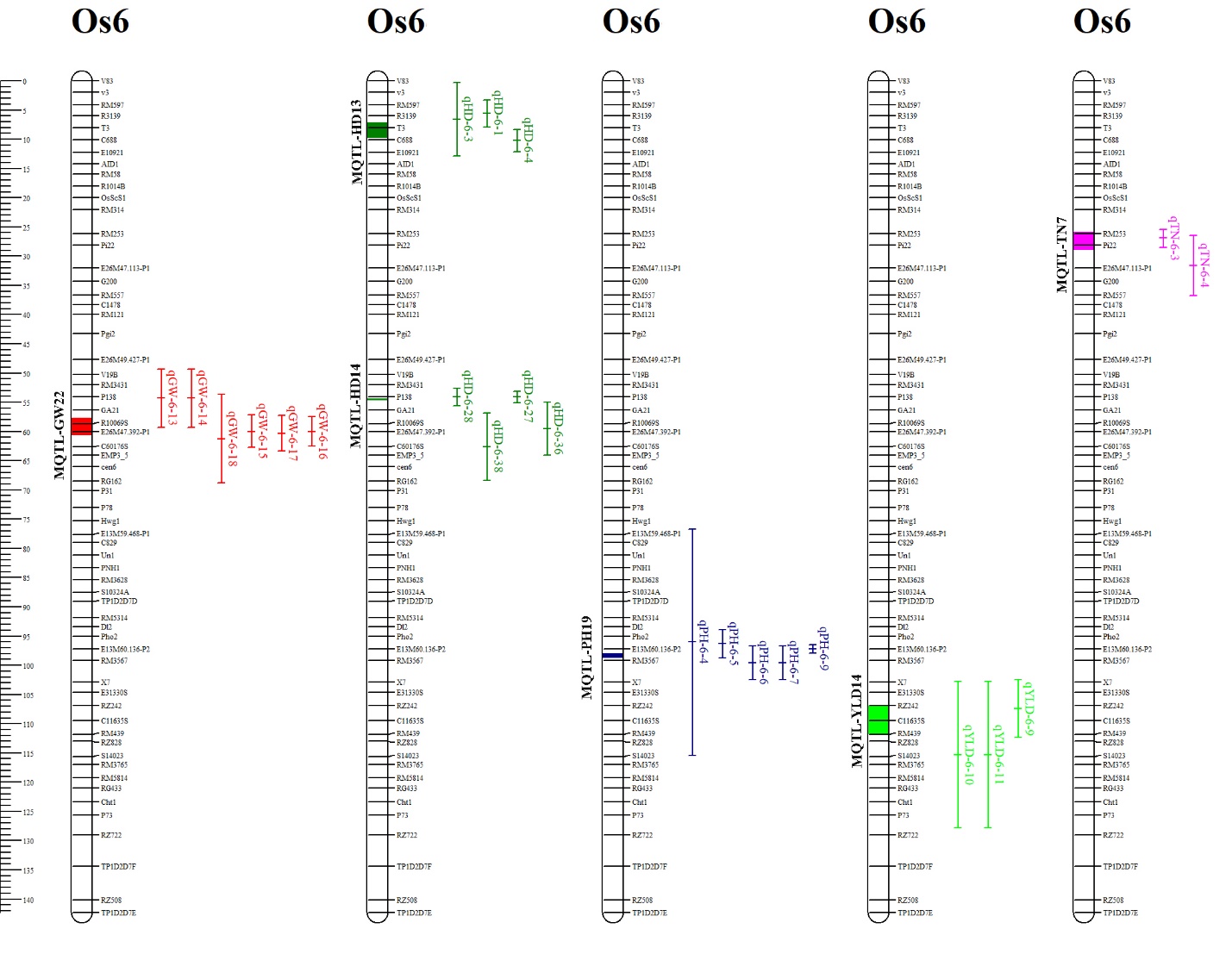

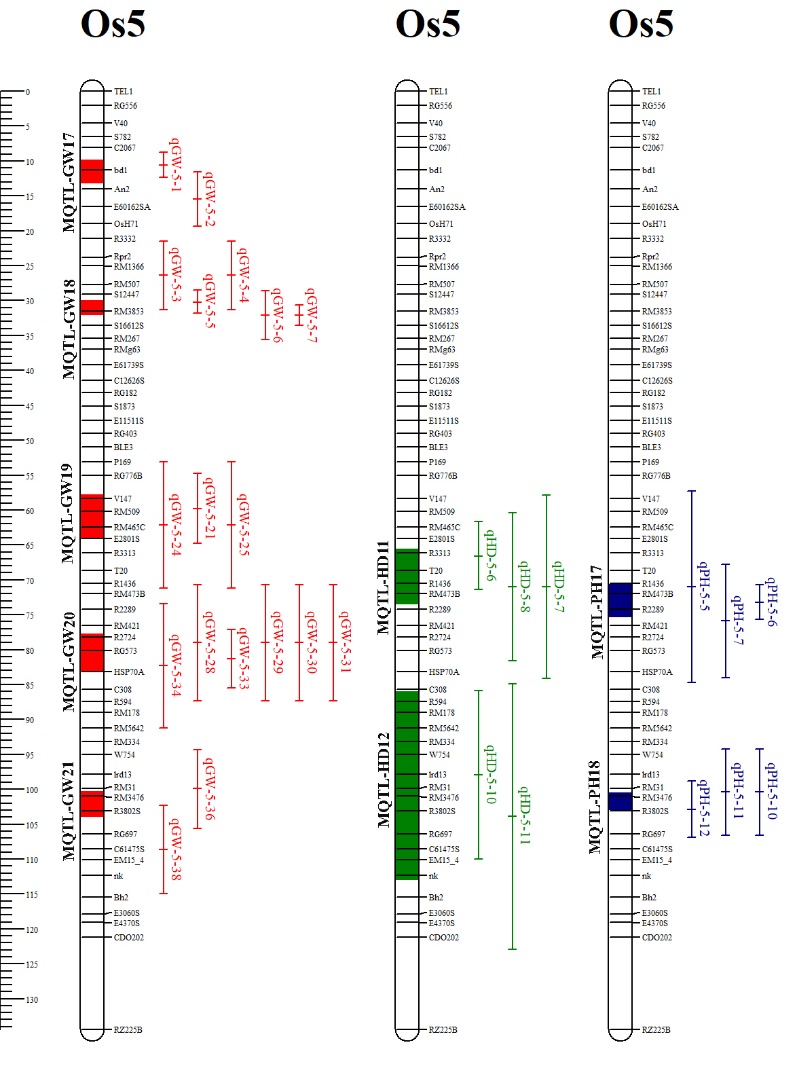


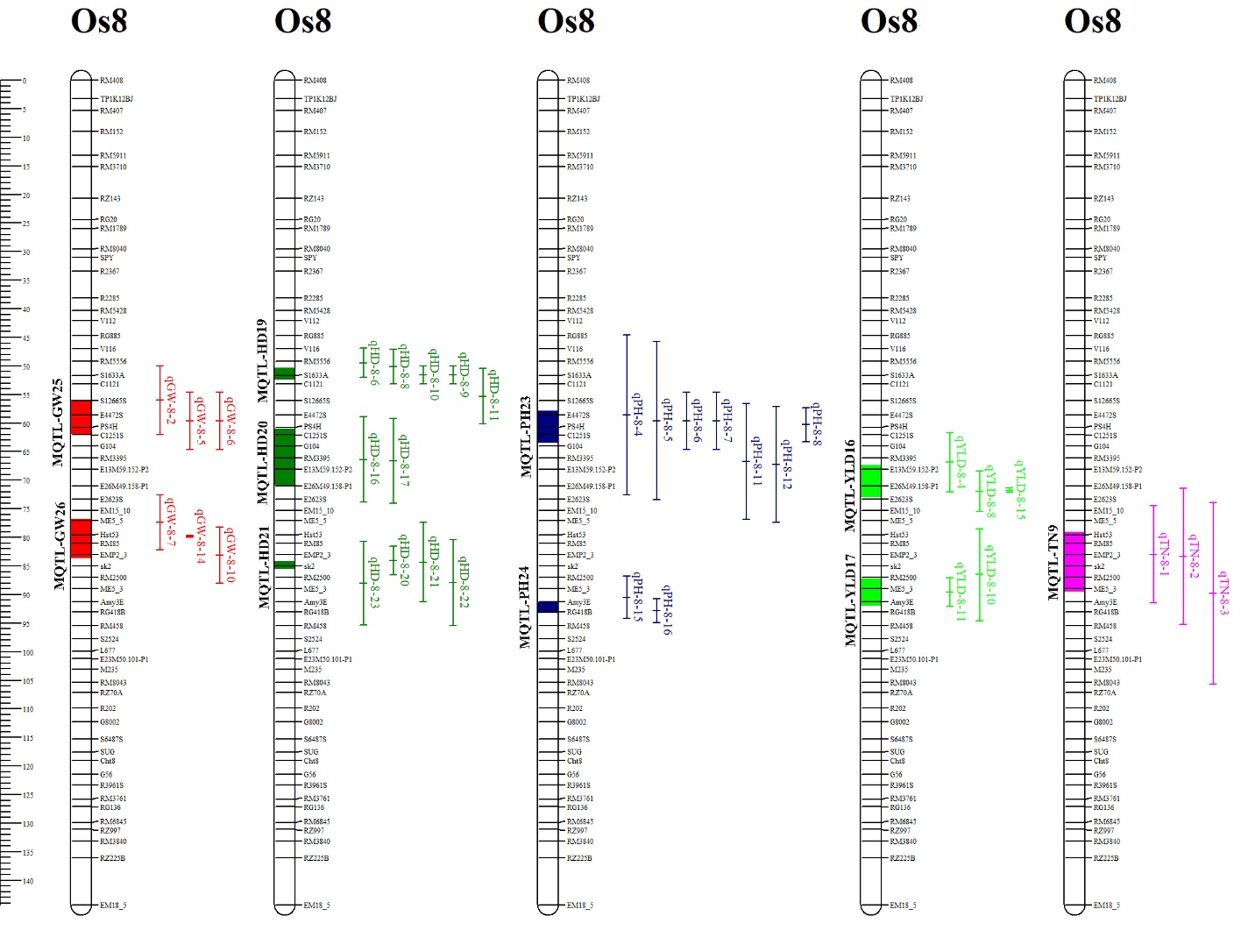

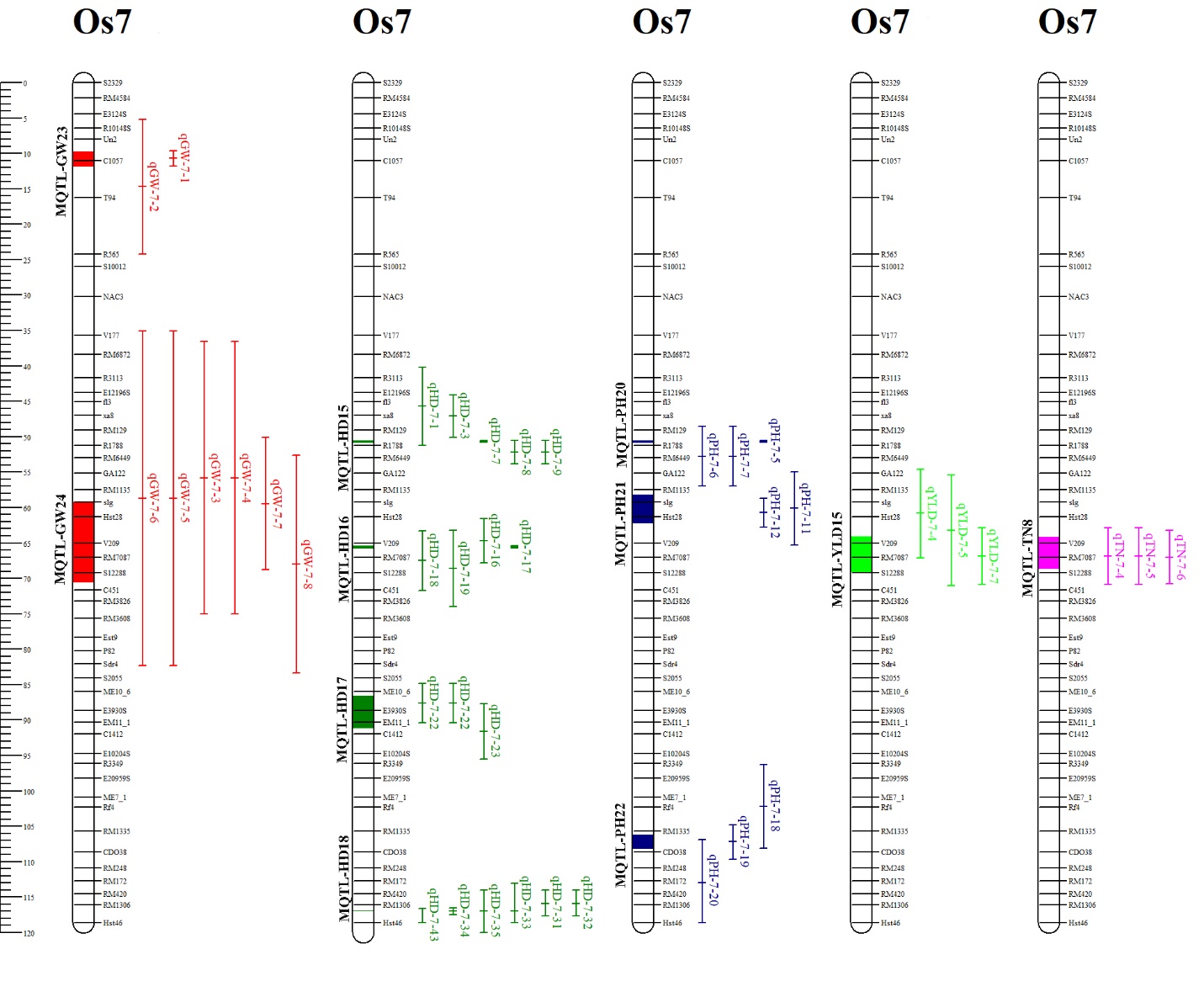


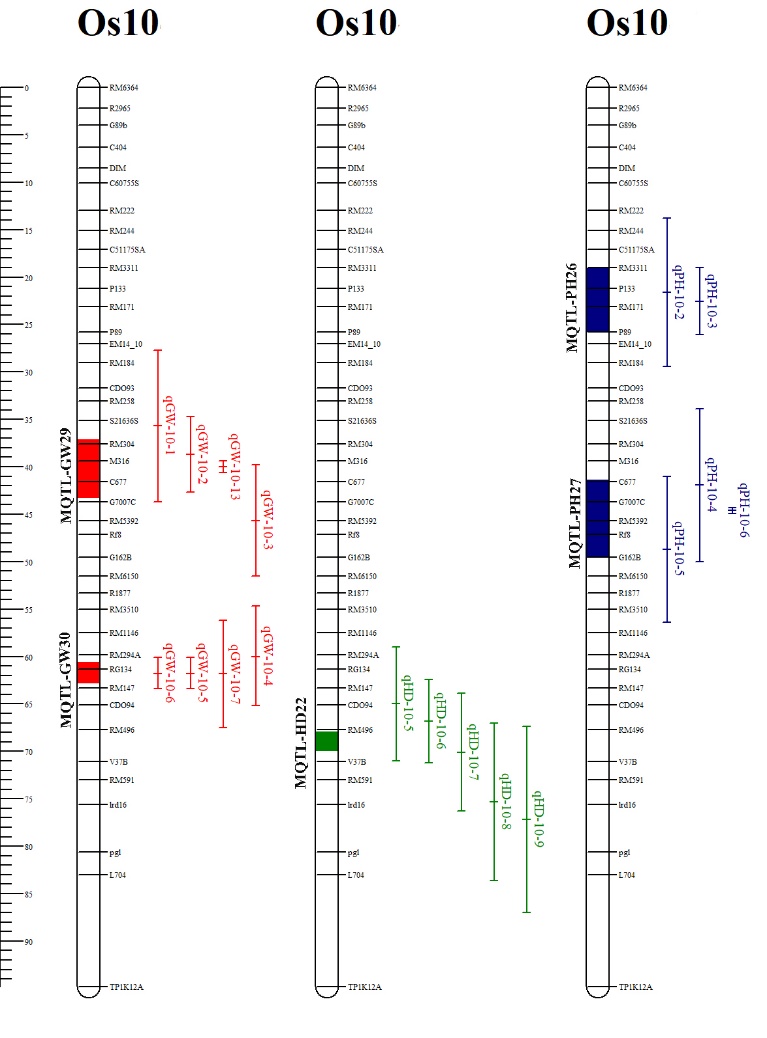

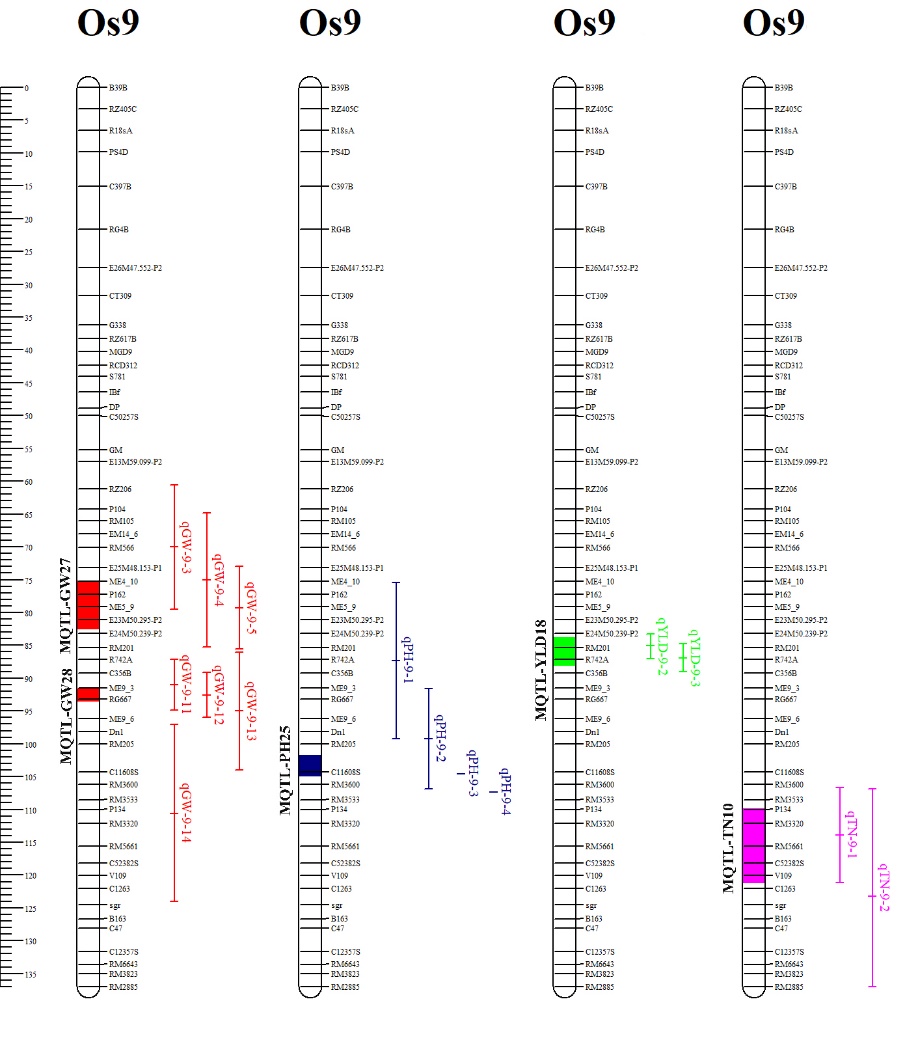


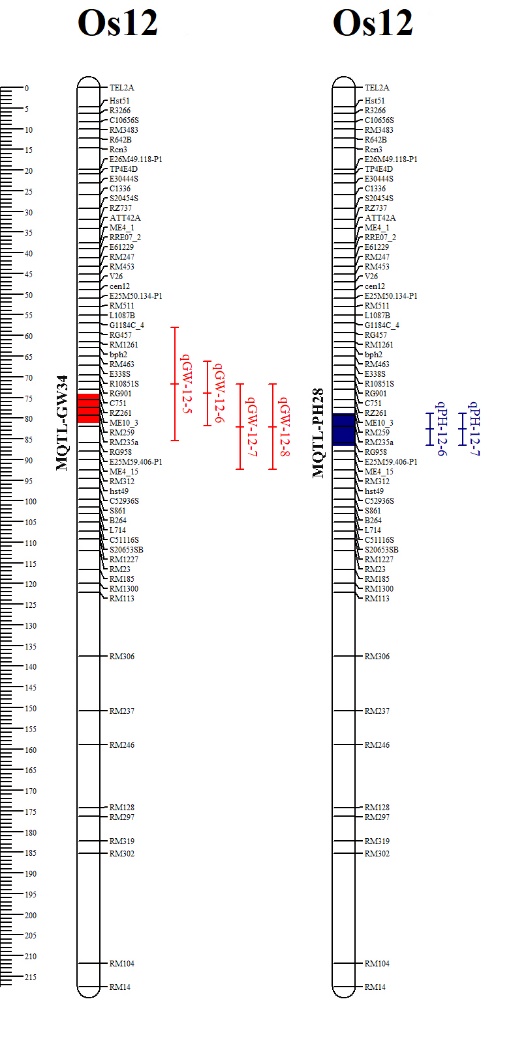

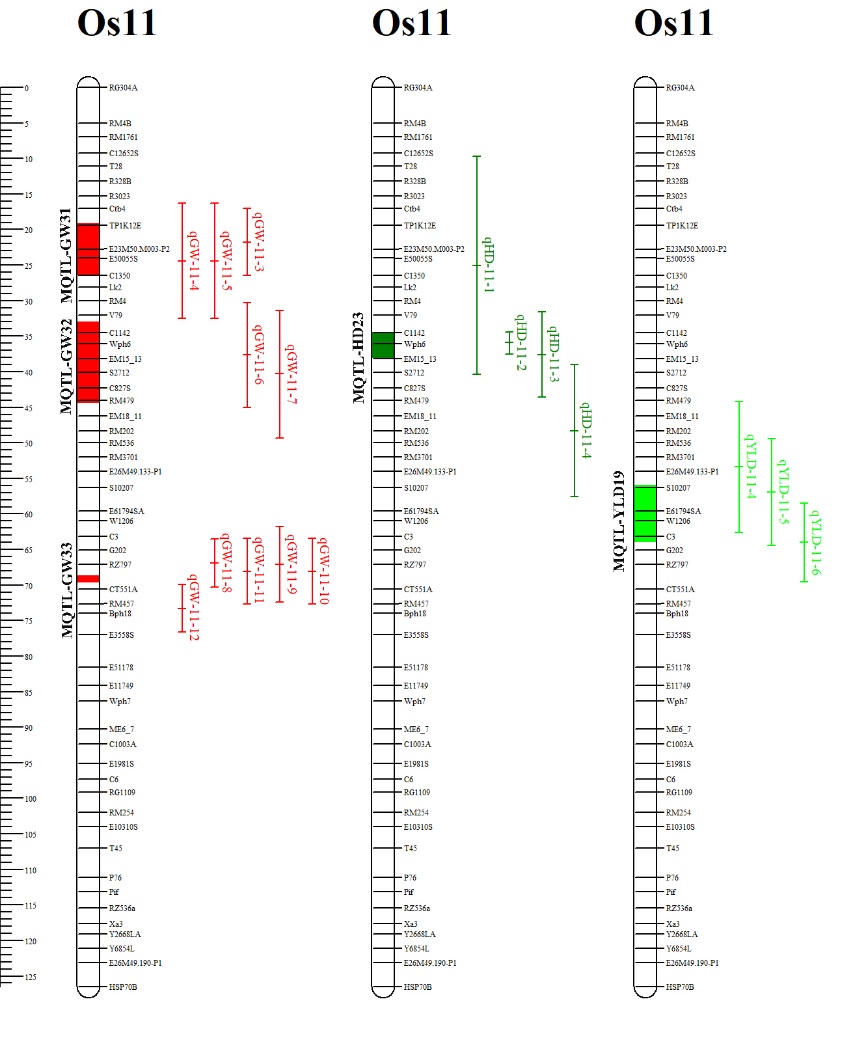


Fig. S1. The chromosomal location of MQTLs and initial QTLs for YLD, HD, PH, GW and TN on 12 chromosomes of rice. MQTLs are shown on each chromosome and the lines on the right side of chromosomes indicate the CI of initial QTLs with 95% confidence intervals. Each color represents a specific trait; GW, HD, PH, YLD and TN are presented in red, dark green, blue, light green and purple, respectively. The markers are shown on the right side of chromosomes. The genetic distance (cM) is indicated on the left side of each chromosome.
