# Supplementary material for: Genome wide screening and comparative genome analysis for Meta-QTLs, ortho-MQTLs and candidate genes controlling yield and yield-related traits in rice
Source: BMC Genomics. 2020 Apr 10;21:294. doi: 10.1186/s12864-020-6702-1 (PMC7146888; doi:10.1186/s12864-020-6702-1)
Supplement: Supplementary file 5 — Additional file 5. The genomic position of MQTLs co-located at GWAS significant results related to GW and HD (Rice SNP-Seek Database) on all chromosomes of rice. [file 12864_2020_6702_MOESM5_ESM.docx]

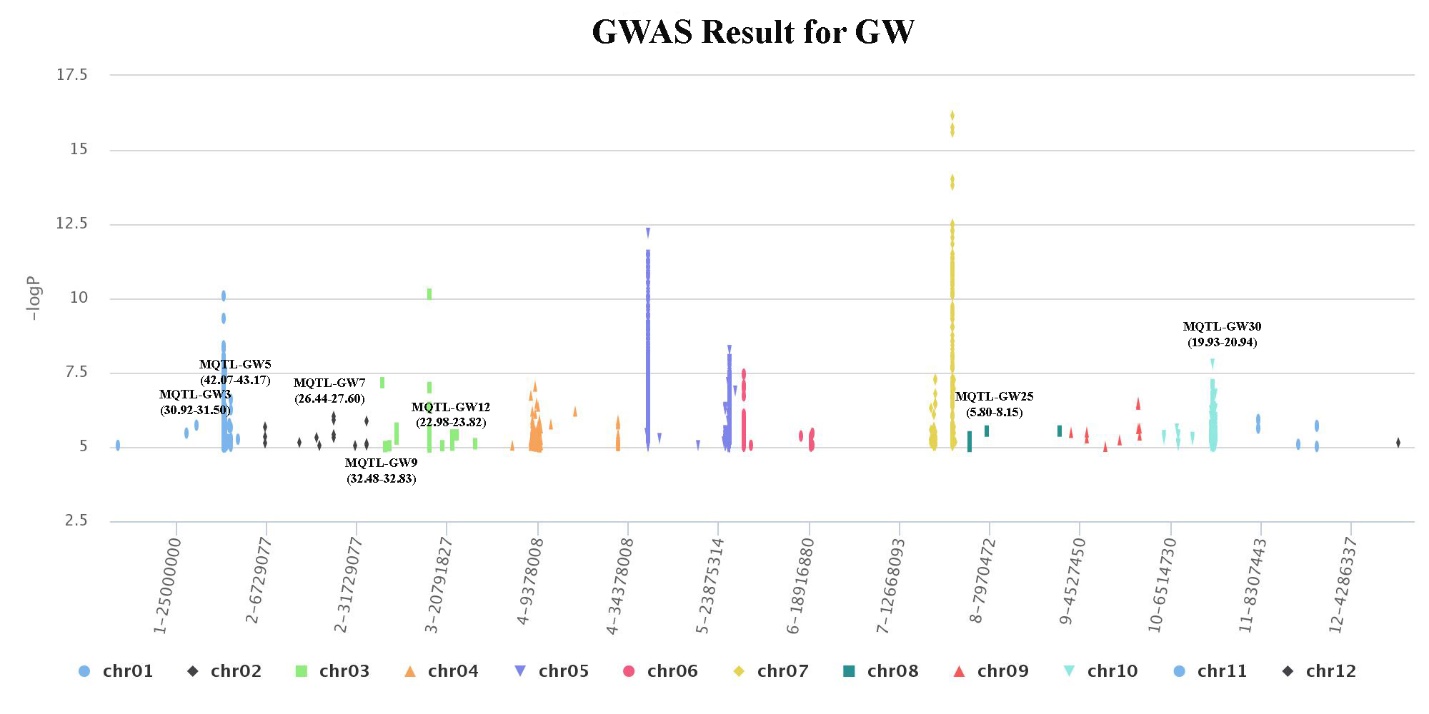


Fig. S2. GWAS results of Grain Weight (GW) in rice through Rice SNP-Seek Database on all chromosomes of rice. The genomic position of nearly and precisely co-located MQTLs are indicated.


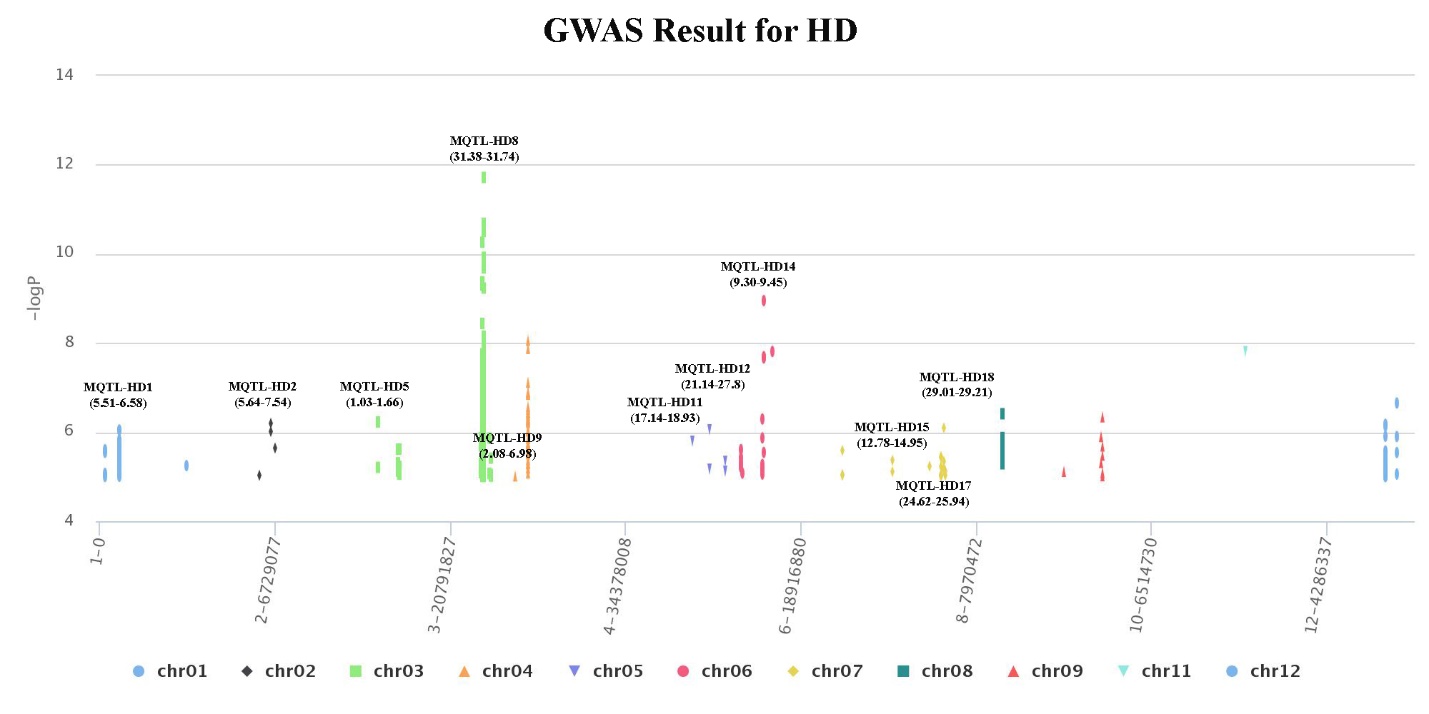


Fig. S3. GWAS results of heading date (HD) in rice through Rice SNP-Seek Database on all chromosomes of rice. The genomic position of nearly and precisely co-located MQTLs are indicated.
